# The GTPase domain of gamma-tubulin is required for normal mitochondrial function and spatial organization

**DOI:** 10.1038/s42003-018-0037-3

**Published:** 2018-05-03

**Authors:** Lisa Lindström, Tongbin Li, Darina Malycheva, Arun Kancharla, Helén Nilsson, Neelanjan Vishnu, Hindrik Mulder, Martin Johansson, Catalina Ana Rosselló, Maria Alvarado-Kristensson

**Affiliations:** 1 Molecular Pathology, Department of Translational Medicine, Lund University, Skåne University Hospital Malmö, 20502 Malmö, Sweden; 2 AccuraScience LLC, 5721 Merle Hay Road, Suite #16B, Johnston, IA 50131 USA; 3 Pathology, Department of Translational Medicine, Lund University, Skåne University Hospital Malmö, 20502 Malmö, Sweden; 4 Unit of Molecular Metabolism, Lund University Diabetes Centre Malmö, 20502 Malmö, Sweden

## Abstract

In the cell, γ-tubulin establishes a cellular network of threads named the γ-string meshwork. However, the functions of this meshwork remain to be determined. We investigated the traits of the meshwork and show that γ-strings have the ability to connect the cytoplasm and the mitochondrial DNA together. We also show that γ-tubulin has a role in the maintenance of the mitochondrial network and functions as reduced levels of γ-tubulin or impairment of its GTPase domain disrupts the mitochondrial network and alters both their respiratory capacity and the expression of mitochondrial-related genes. By contrast, reduced mitochondrial number or increased protein levels of γ-tubulin DNA-binding domain enhanced the association of γ-tubulin with mitochondria. Our results demonstrate that γ-tubulin is an important mitochondrial structural component that maintains the mitochondrial network, providing mitochondria with a cellular infrastructure. We propose that γ-tubulin provides a cytoskeletal element that gives form to the mitochondrial network.

## Abstract (web summary)

Lisa Lindström et al. find that the gamma-tubulin cellular network is required to maintain mitochondrial function and organization in the cell. Knockdown of gamma-tubulin or loss of its GTPase domain disrupts the mitochondrial network and alters respiratory capacity and expression of mitochondrial genes.

## Introduction

Cellular compartmentalization is a hallmark of eukaryotic cells and transport between compartments is required to maintain cellular homeostasis and energy production^1^. This cellular organization is based on the interaction between actin filaments, microtubules and intermediate filaments with cellular organelles, such as mitochondria^1^. Both actin filaments and microtubules can dynamically assemble and disassemble polar filaments that contribute to cell polarity and cell division, whereas intermediate filaments form non-polar structures that are disassembled during mitosis^2^.

An important regulator of microtubule dynamics during cell division is the protein γ-tubulin^3, 4^. We and others have recently reported that γ-tubulin forms a cellular meshwork of γ-strings^5–7^ and γ-tubules^8^. While γ-tubules are polar cytosolic filaments within the γ-string meshwork, γ-strings are detected in both the cytoplasm and the nucleus and are formed of non-polar protein threads that cross the double membrane of the nuclear envelope. The γ-string meshwork forms a boundary around chromatin, which coordinates cytosolic and nuclear events during mitosis by assuring that a nuclear envelope forms around daughter chromosomes^5^. Furthermore, the γ-string meshwork formed by the C-terminal DNA-binding region of γ-tubulin forms a cytosolic network as well^9^. These observations together suggest that the γ-tubulin meshwork may be a dynamic network that contributes to cellular homeostasis.

In the present study, we characterize the dynamics of the γ-tubulin meshwork and its implication in cellular homeostasis. We show that γ-strings are a mitochondrial structural component that associates with both mitochondrial DNA and membranes. In addition, we demonstrate that the GTPase domain of γ-tubulin facilitates the organization of a mitochondria-associated γ-string meshwork and that γ-tubulin depletion disrupts the meshwork. Our findings highlight an essential role for γ-tubulin in mitochondrial structure and ultimately mitochondrial function.

## Results

### C-terminal γ-tubulin^336–451^ associates with mitochondria

**Fig. 1:**
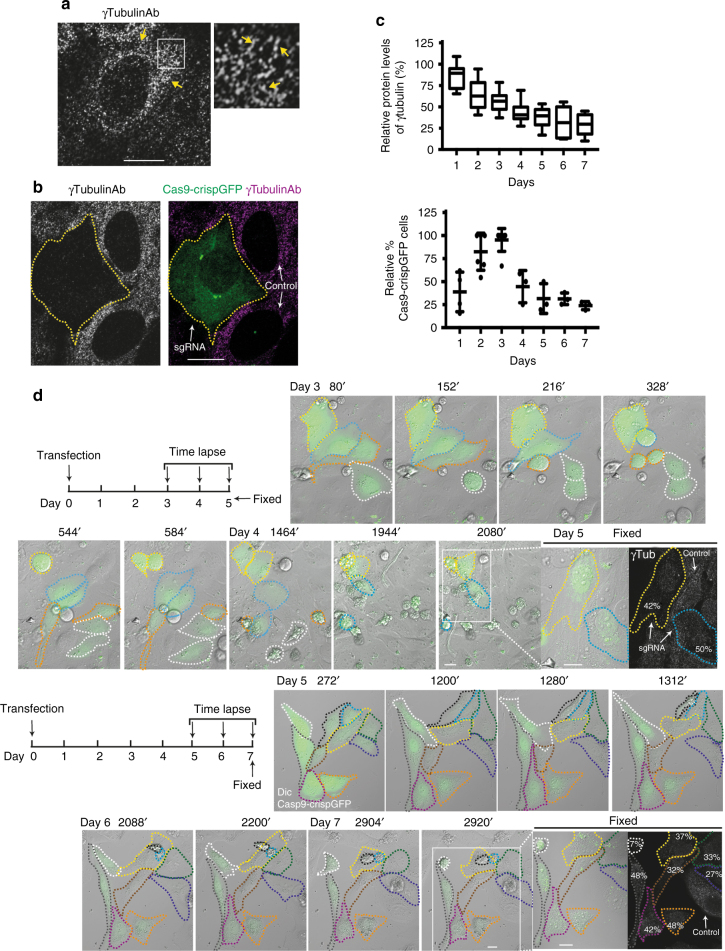
γ-Tubulin forms protein strings and γ-tubulin knockdown is cytotoxic. **a**, **b** Confocal images of fixed U2OS or U2OS expressing *γTubulin* sgRNA (Cas9-crispGFP; green) that were immunostained with an anti-γ-tubulin (γTubulinAb) antibody originated in mouse. **a** The white box shows the magnified area displayed in the inset. **a**, **b** Yellow and white arrows show γ-strings or the indicated cell, respectively (*N* = 5). **c** U2OS expressing *γTubulin* sgRNA (Cas9-crispGFP) at day 0 were incubated for the indicated time before fixation. Cells were stained as in **a**. Within samples, quantification of γ-tubulin was done with ImageJ software by comparison of immunofluorescently labelled γ-tubulin in cells expressing Cas9-crispGFP with non-expressing cells (control; *N* = 7–11 cells). Graph represents the relative percentage of cells that expressed Cas9-crispGFP at the indicated period of time. To adjust for differences in transfection efficiency, the sample containing the largest number of cells expressing Cas9-crispGFP was defined as 100% and values at other time points were compared with that sample (*N* = 3–7). **d** Schematic representation of the time-lapse experiments. Cells were transfected (day 0) and experiments started three or five days after, as indicated. Cell populations were monitored for three days before fixation and subsequently immunostained with an anti-γ-tubulin (γTub) antibody that originated in mouse. The levels of endogenous γ-tubulin were determined as in **c**. The differential interference contrast (DIC)/fluorescence images show time-lapse series from U2OS cells expressing Cas9-crispGFP (green; dashed lines). Images were collected every 8 min. Top images show four cells and their respective daughter cells (yellow, blue, orange and white) before dying (white, orange, yellow and blue). Bottom images show ten cells that either remained in interphase (yellow, light blue, dark blue, orange, black, grey, brown, magenta, green, and white) or died (black, light blue, and white) during the course of the experiment. White boxes show the live cells displayed in the insets. Insets show γ-tubulin in fixed cells (Fixed) expressing Cas9-crispGFP. Numbers in images indicate the remaining protein levels of γ-tubulin relative to control in the indicated cells (*N* = 3). Scale bars are 10 μm in images. Please, see Supplementary Fig. 1

We found that in close vicinity to the nuclear envelope, endogenous γ-tubulin formed a network of strings, γ-strings, which grew from the nuclear compartment and towards the plasma membrane (Fig. 1a). The immunofluorescence staining of γ-strings was abolished following gene editing, using a single-guide (sg)RNA targeting the γ-tubulin genes, *TUBG1* and *TUBG2* (*γTubulin* hereafter, unless a specific isoform is referenced) , demonstrating that γ-strings consist of γ-tubulin (Fig. 1b). Notably, we found that sgRNA-induced reduction of γ-tubulin was cytotoxic when γ-tubulin protein levels dropped below 50% (Fig. 1c). After *γTubulin* sgRNA transfection, cells divided during the subsequent three to four days. Thereafter, cells remained in interphase for several days before dying (Fig. 1d). Immunofluorescence staining of *γTubulin* sgRNA expressing cells with an anti-cytochrome c antibody and a chromatin dye, showed the reduction of γ-tubulin expression caused the mitochondrial release of cytochrome c and chromatin condensation, both apoptotic markers (Supplementary Fig. 1)^10^.

**Fig. 2:**
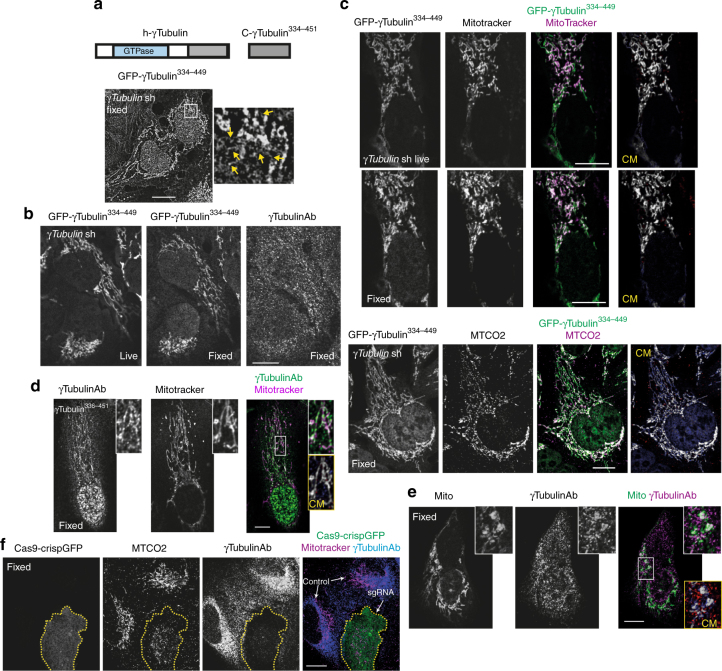
Endogenous cytosolic γ-tubulin associates with mitochondria. **a** Structure of human wild-type γ-tubulin (h-γTubulin) and the γ-tubulin C terminus (C-γTubulin^336–451^), depicting the GTPase domain and the C-terminal region of γ-tubulin. U2OS cells stably expressing *γTubulin* shRNA and sh-resistant GFP-γ-tubulin_resist_^334-449^ fragment were imaged by structured illumination microscopy. The yellow arrows show γ-strings. **b**, **c** Confocal fluorescence images of fixed or live U2OS cells stably expressing *γTubulin* shRNA and GFP-γ-tubulin_resist_^334–449^. Mitochondria were stained with the fluorescent dye MitoTracker or with the mitochondrial marker cytochrome c oxidase subunit II (MTCO2) and the total pool of γ-tubulin was immunofluorescence stained with an anti-γ-tubulin (γTubulinAb) antibody originated in rabbit. **c** Co-localization pixel maps (CM) of the red and green (blue) channels of images are shown. White areas denote colocalized pixels between channels (MitoTracker (life), Person’s *R* = 0.7, fraction of red (MitoTracker) overlapping blue (GFP-γ-tubulin_resist_^334–449^) M1 = 1.0, fraction of blue overlapping red M2 = 0.9; MTCO2, Person’s *R* = 0.5, fraction of red (MTCO2) overlapping blue (GFP-γ-tubulin_resist_^334–449^) M1 = 1.0, fraction of blue overlapping red M2 = 0.8). **d** Confocal fluorescence images of U2OS cells stably expressing γ-tubulin^336–451^. The mitochondria were stained with MitoTracker and the total pool of γ-tubulin with a γTubulinAb originated in rabbit. **e** Immunofluorescent staining of endogenous γ-tubulin in U2OS cells transiently expressing pmTurquoise2-mito (mito) with a γTubulinAb originated in rabbit. **d**, **e** Co-localization pixel maps (CM) of the red and green (blue) channels of the magnified areas displayed in the inset (the yellow box). White areas denote colocalized pixels between channels (**d**, MitoTracker, Person’s *R* = 0.7, fraction of red (MitoTracker) overlapping blue (γTubulinAb) M1 = 1.0, fraction of blue overlapping red M2 = 0.9; **e**, Mito, Person’s *R* = 0.5, fraction of red (γTubulinAb) overlapping blue (Mito) M1 = 0.9, fraction of blue overlapping red M2 = 1.0). **a**, **d**, **e** The white box shows the magnified areas displayed in the inset. **f** Fixed U2OS cells transiently expressing *γTubulin* sgRNA (Cas9-crispGFP) were immunofluorescence stained with an anti-MTCO2 antibody and a γTubulinAb originated in mouse. (**a**–**f**) The figure shows representative images from at least six experiments. Scale bars are 10 μm in images. Please, see Supplementary Fig. 2

In contrast to cells expressing *γTubulin* sgRNA that undergo apoptosis (Fig. 1c, d), we instead used stable *γTubulin* shRNA expressing cells (*γTubulin*sh-U2OS). The *γTubulin* shRNA expression reduced the endogenous γ-tubulin pool by ~50% (Supplementary Fig. 2a)^5, 11^, and we compensated for this reduction by stably co-expressing a sh-resistant human GFP-C-terminal region (residues 334–449, GFP-γ-tubulin^334–449^) in U2OS cells (*γTubulin*sh-U2OS-γ-tubulin^334–449^; Fig. 2a, Supplementary Fig. 2a). We found that the endogenous γ-strings (Fig. 1a) were similar to those structures formed by *γTubulin*sh-U2OS-γ-tubulin^334–449^, as shown by the immunofluorescence staining of *γTubulin*sh-U2OS-γ-tubulin^334–449^ with an anti-γ-tubulin antibody (Fig. 2b). Super-resolution microscopy showed that various GFP-γ-strings^334–449^ merged forming a dense protein meshwork (Fig. 2a).

By associating with the nuclear envelope, γ-strings give support to the double nuclear membrane structure and connect the cytoplasm with the chromatin^5^. Similar to the nuclear compartment, mitochondria contains both DNA and is surrounded by a double membrane^12^. Recent work demonstrates an association of γ-tubulin with mitochondrial membranes^13^. In addition, the γ-tubulin’s DNA binding motif is encoded in the γ-tubulin C terminus^14^. Thus, we hypothesized that γ-strings may connect the cytoplasm with the mitochondrial DNA (mtDNA). Consequently, the γ-string meshwork in close proximity to the nuclear envelope, formed by either endogenous γ-tubulin (Fig. 1a) or GFP-γ-tubulin^334–449^ (Fig. 2a, b), looked similar in structure, and its positioning resembled the mitochondrial network formed in close proximity to the nuclear envelope^15^. To analyze a possible association between γ-strings and mitochondria, we used immunofluorescence to stain *γTubulin*sh-U2OS-γ-tubulin^334-449^ with a fluorescent dye that identified mitochondria in live cells (MitoTracker, Fig. 2c), or with an antibody that recognized the mitochondrial marker cytochrome c oxidase subunit II (MTCO2; Fig. 2c). We also expressed γ-tubulin^336-451^ in U2OS to ascertain that the GFP did not affect the cellular positioning of the C-terminal region (Fig. 2b, d, Supplementary Fig. 2b). In addition, in U2OS cells, we ectopically expressed the pmTurquoise2-tagged mitochondrial-targeting signal from cytochrome c oxidase subunit VIII A (amino acids 1–29; Fig. 2e, Supplementary Fig. 2c), which from now on will be referred as mito^15^. Confocal microscopy confirmed that endogenous γ-strings, recombinant GFP-γ-strings^334–449^, and γ-tubulin^336–451^ formed a dense meshwork in mitochondria-rich areas (Fig. 2c–e). Finally, sgRNA mediated reduction of γ-tubulin disrupted the mitochondrial network (Fig. 2f). These data suggest that γ-tubulin affects the morphology of the mitochondrial meshwork.

### γ-Tubulin is a mitochondrial protein

**Fig. 3:**
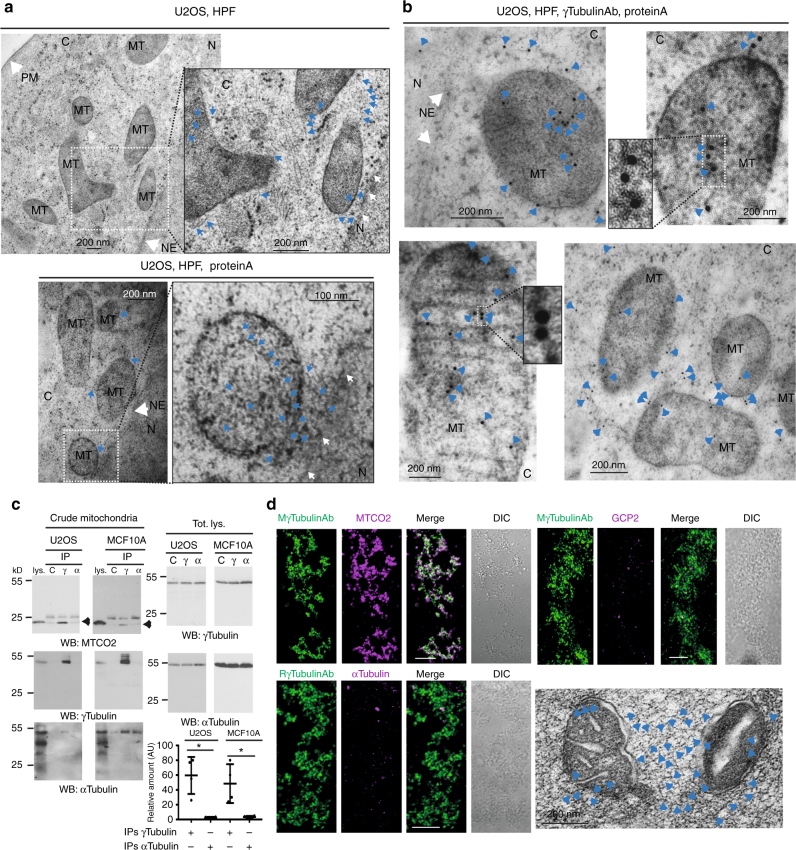
γ-Strings are associated with mitochondria. **a**, **b** Immunoelectron microscopy detection of endogenous γ-tubulin using three different conditions in high-pressure frozen (HPF) U2OS cells: first, no antibody (**a**), second, gold conjugated protein A (**a**) and third, an anti-γ-tubulin antibody originated in rabbit, and gold conjugated protein A (**b**, γTubulinAb). Images show the plasma membrane (PM), the nuclear envelope (NE), cytosol (C), mitochondria (MT) and nucleus (N) of a U2OS cell. Blue arrows show γ-strings and arrowheads show immunolabelled γ-strings. White arrows show the nuclear envelope or the plasma membrane, as indicated. White dashed boxes show the magnified areas displayed in the inset (*N* = 5). **c** The crude mitochondria fraction from U2OS and MCF10A cells was biochemically prepared. Each sample was subjected to immunoprecipitation (IP) with a control (C), an anti-γ-tubulin (γ; originated in mouse) or an anti-α-tubulin (α) antibody, as indicated, and developed by Western blotting (WB) with an anti-MTCO2 antibody (top, arrowhead), and then reprobed with γ-tubulin (originated in rabbit) and α-tubulin. Aliquots of the cytosolic lysates used in the immunoprecipitations were run as loading controls (lys. and Total lys.) and analyzed by Western blotting. Graph shows the mean content of γ-tubulin and α-tubulin found in their respective immunoprecipitates in the mitochondrial crude fraction. To adjust for differences between WBs, the protein content in control immunoprecipitates was defined as 1 and values of the other immunoprecipitates were compared with that level (mean ± SD; *N* *=* 4, *P < 0.05). **d** The biochemically isolated crude mitochondria fraction from a MCF10A cell population was fixed and immunostained with anti-γ-tubulin originating in mouse (M) or rabbit (R) and anti-MTCO2, anti-α-tubulin or anti-GCP2 antibody, as indicated. Scale bars are 10 μm in images. The electron microscopy image shows mitochondria in the crude mitochondria fraction. Arrowheads show γ-strings between and in mitochondria (*N* = 4). Please, see Supplementary Fig. 3

Interestingly, despite evidence that the mitochondrial proteome is derived from endosymbiotic bacteria^12^, most mitochondria lack the expression of the γ-tubulin homologue FtsZ^16^, suggesting that γ-tubulin may replace FtsZ function in mitochondria. To confirm a possible role of endogenous γ-tubulin in mitochondria, we investigated the distribution of endogenous γ-tubulin by immunoelectron microscopy of high-pressure frozen U2OS cells (Fig. 3a, b). As γ-tubulin is distributed throughout the cell, we optimized the antibody concentration so that cellular structures were only partially stained. We found that mitochondria were connected to each other, and to the nuclear membrane by protein strings, which were 4–6 nm in diameter (Fig. 3a, b). Furthermore, a γ-tubulin antibody recognized these strings (Fig. 3b)^5^ and the immunostaining was negated following *γTubulin* sgRNA expression, confirming the specificity of the antibody (Supplementary Fig. 3a). Finally, the γ-tubulin antibody was able to stain γ-strings both inside and outside the mitochondria (Fig. 3b). Together, the data presented here implies that γ-strings form a network that harbours mitochondria.

Previous work demonstrates that microtubules are necessary for both mitochondrial activity and mitochondrial intracellular motor-driven transport^17, 18^. To compare the amount of mitochondria-associated γ-tubulin with the amount of mitochondria-associated microtubules, we analyzed immunoprecipitates of endogenous γ- and α-tubulin from the crude mitochondria fraction. The immunoprecipitated complexes highlighted an association of γ-tubulin with the mitochondrial membrane marker MTCO2 in U2OS and MCF10A cells (Fig. 3c, Supplementary Fig. 3b). Moreover, we noticed that in comparison to α-tubulin, γ-tubulin was highly enriched in the crude mitochondrial fraction (Fig. 3c), confirming the presence of a mitochondria-associated γ-tubulin pool that is not microtubule related. Accordingly, Western blot analysis of lysates from mitochondria from MCF10A cells prepared by density gradient centrifugation using Percoll confirmed the presence of γ-tubulin in the mitochondria (Supplementary Fig. 3c).

In the cytoplasm, γ-tubulin together with various γ-tubulin complex proteins (GCP) is able to nucleate microtubules by forming the γ-tubulin ring shaped complex (γTURC)^3, 4, 19^. To study a possible association of the γTURC with the mitochondria, we performed immunofluorescent staining of the isolated crude mitochondria fraction from MCF10A cells, which confirmed that, in comparison to α-tubulin and GCP2, γ-tubulin is enriched in mitochondria (Fig. 3d). Finally, electron microscopy analysis of the isolated crude mitochondrial fraction showed the presence of γ-strings between and inside mitochondria (Fig. 3d). Thus, the association of γ-strings with mitochondrial membranes suggests that γ-tubulin forms a membrane-associated meshwork that provides mitochondria with a structural scaffold.

### γ-Tubulin binds to mitochondrial DNA

**Fig. 4:**
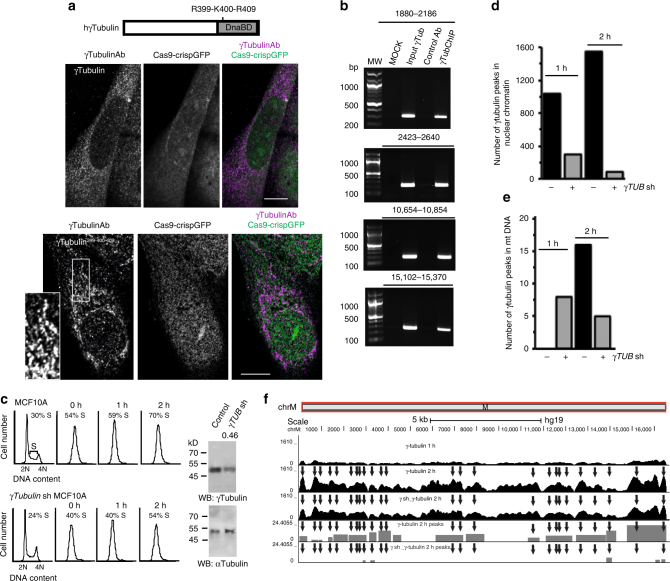
γ-Tubulin binds to mitochondrial DNA. **a** Structure of human wild-type γ-tubulin and the γ-tubulin DNA-binding domain (DnaBD), depicting residues R399, K400 and R409 in the nuclear localization signal of γ-tubulin. Confocal fluorescence microscopy of fixed U2OS stably expressing *γTubulin* sgRNA (Cas9-crispGFP) and co-expressing a *γTubulin* sgRNA resistant transcript (γTubulin) or a mutant form, γTubulin^R399A-K400A-R409A^ (γTubulin^399-400-409^). The recombinant proteins were immunostained with an anti-γ-tubulin antibody (γTubulinAb) that originated in mouse. **b** U2OS cells were analyzed by ChIP using an anti-γ-tubulin antibody (γTubChIP) that originated in rabbit. PCR primers amplified the indicated regions of the mitochondrial DNA (*N* = 3). **c** MCF10A and *γTubulin* shRNA stably expressing MCF10A (γ*Tubulin* sh MCF10A) cells were synchronized in early S-phase (0 h) by double thymidine block and released for 1 h and 2 h. Cell cycle progression was monitored by determining the DNA content of cells with a nucleocounter (graphs; *N* = 3). Total lysate from MCF10A (Control) and MCF10A cells stably expressing *γTubulin* shRNA (γ*Tub* sh) were analyzed by Western blot (WB) for the expression of endogenous γ-tubulin. An α-tubulin loading control is shown (*N* = 3). The number on the WB indicates the level of depletion of γ-tubulin relative to control. To adjust for differences in protein loading, the protein concentration of γ-tubulin was determined by its ratio with endogenous α-tubulin. The protein ratio in control extracts was set to 1. **d**, **e** To map the location of γ-tubulin in the chromatin of MCF10A and of MCF10A cells stably expressing *γTubulin* shRNA (γ sh), we sequenced the DNA associated with chromatin immunoprecipitates from γ-tubulin. Immunoprecipitations were performed in early S-phase synchronized cell populations using an anti-γ-tubulin antibody. Graphs show the number of binding sites (peaks called) found in the human genome (**d**) or mitochondrial chromosome (**e**) to which γ-tubulin binds at the indicated period of time. **f** The graphs show ChIP-seq analysis of γ-tubulin distribution on mitochondrial chromosome (ChrM). The entire chromosome M is presented. Black arrows indicate areas loaded with γ-tubulin. In grey is the schematic representation of the called peaks (*N* = 2). Please, see Supplementary Fig. 4

**Fig. 5:**
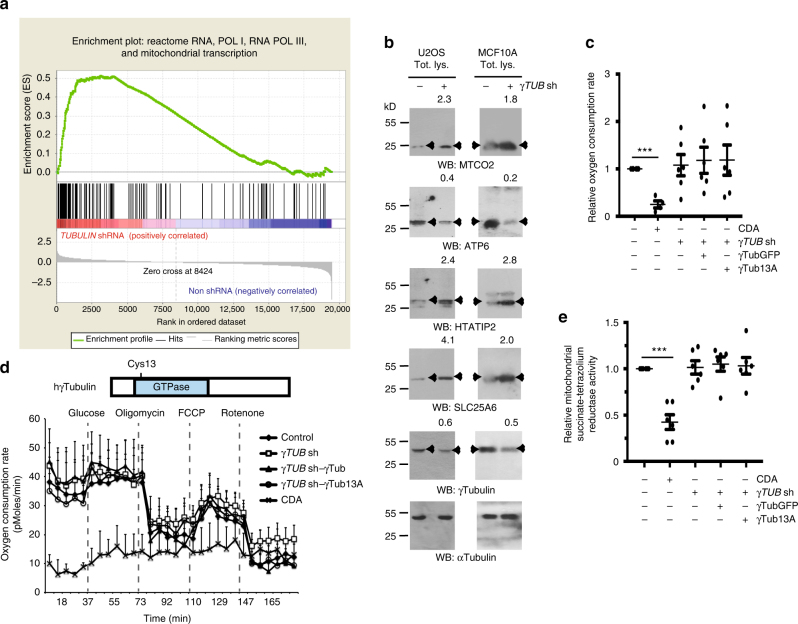
The γ-tubulin meshwork controls mitochondrial activity. **a** GSEA of mitochondrial-upregulated gene set performed on a ranked gene list of differentially expressed genes between synchronized MCF10A (non shRNA) and *γTubulin*sh-MCF10A (*Tubulin* shRNA) cells. **b** Western blots (WB) show total lysates (Tot. lys.) of U2OS or MCF10A cells that stably expressed *γTubulin* shRNA (γ*TUB* sh) using the indicated antibodies. Anti-α-tubulin antibody was used as loading control (*N* = 3). Arrowheads indicate proteins whose expression is affected by the expression of *γTubulin* shRNA. The numbers on the WBs indicate variations in MTCO2, ATP6, HTATIP2, SLC25A6, and γ-tubulin expression relative to *γTubulin* shRNA non-expressing cells, as indicated. To adjust for differences in protein loading, the protein concentration of the various proteins was determined by their ratio with α-tubulin for each sample. The protein ratio in control extracts was set to 1. **c** The mean values of the relative basal oxygen consumption was determined using Seahorse analyser in U2OS cells, U2OS cells stably expressing *γTUB* sh and stably co-expressing GFP-γ-tubulin_resist_ (γTubGFP) or GFP-A^13^γ-tubulin_resist_ (γTub13A) and U2OS cells pre-treated with CDA for 2 h. Note that CDA is present during the Seahorse assay, which takes 3 h. The data were normalized to the total number of cells. The oxygen consumption rate activity of U2OS cells was set as 1, and relative activities were calculated (mean ± SEM; *N* = 4–6, ***P < 0.001). **d** Structure of human wild-type γ-tubulin (h-*γTubulin*), depicting residue Cys13 in the GTPase domain of γ-tubulin. Graph shows seahorse assay of the respiratory capacity after addition of glucose mixture (20 mM glucose, 20 μg/ml insulin), 4 μg/ml oligomycin, 1 μM FCCP and 0.5 μM Rotenone in 28 × 10^3^ of the indicated cells. Note the very low basal oxygen consumption rate of CDA-treated U2OS cells (mean ± SEM; *N* *=* 4–6). **e** WST-1 assay showing the metabolic activity of U2OS cells transfected with the indicated construct or after 4 h CDA pre-treatment (mean ± SEM; *N* = 6, ***P < 0.001)

A nuclear localization signal (NLS) mediates γ-tubulin translocation to the nucleus^14^. To study the effect of mutations in the γ-tubulin NLS, we stably co-expressed *γTubulin* sgRNA (depleted the endogenous γ-tubulin pool), and either a wild-type sg-resistant human *TUBG1* or a sg-resistant *TUBG1* containing a mutated NLS. In comparison to cells regularly expressing γ-tubulin, we found that mutations of R399A, K400A, and R409A in the NLS^14^ caused the formation of tubular structures (Fig. 4a, Supplementary Fig. 4), suggesting that the reduced import of γ-tubulin to the nuclear compartment enhances the binding of γ-tubulin to mtDNA. To determine whether γ-tubulin binds to mtDNA, we performed a chromatin immunoprecipitation (ChIP) assay using γ-tubulin antibodies and found that endogenous γ-tubulin was present on mtDNA (Fig. 4b). To identify underlying mitochondrial processes associated with γ-tubulin binding to DNA, we synchronized cells in S-phase (Fig. 4c) and mapped the location of γ-tubulin on the mtDNA of MCF10A and of MCF10A cell populations that stably expressed *γTubulin* shRNA (*γTubulin*sh-MCF10A) by sequencing the DNA associated with chromatin immunoprecipitates (ChIP-seq) from γ-tubulin (Fig. 4d–f). Also, we evaluated γ-tubulin’s effect on RNA expression of mitochondrial-related mRNA by massive sequencing of the purified RNA (RNA-seq) from the studied cell populations (Fig. 5a). Differential protein peaks on DNA were called with MACS2 (version 2.1.1)^20^. The identified differential peaks associated with nuclear chromatin were more numerous in γ-tubulin immunoprecipitates from MCF10A cells, than those from *γTubulin*sh-MCF10A cells, suggesting that shRNA-mediated reduction of γ-tubulin protein levels led to a reduced binding of γ-tubulin to genomic DNA (Fig. 4d).

In S-phase, γ-tubulin accumulates in the chromatin of cells^14, 21, 22^. Accordingly, we found an increase in the number of peaks associated with genomic- and mitochondrial DNA as the cells progressed through S-phase (2 h; Fig. 4d–f). The identified differential peaks associated with mtDNA at 2 h were more abundant in γ-tubulin immunoprecipitates from MCF10A cells than those from *γTubulin*sh-MCF10A cells. This indicated that shRNA-mediated reduction of γ-tubulin protein levels led to reduced binding of γ-tubulin to mtDNA (Fig. 4e). However, we found mtDNA in γ-tubulin immunoprecipitates from *γTubulin*sh-MCF10A cells in early S-phase (1 h) that was not found in the ones from MCF10A cells. We think that this may be caused by the limited reduction of γ-tubulin protein levels in *γTubulin*sh-MCF10A cells, which affects γ-tubulin dynamics during S-phase progression. Taken together, the data presented here demonstrate that γ-tubulin binds to mtDNA.

### γ-Tubulin regulates the expression of mitochondrial genes

Given that γ-tubulin associates with both nuclear- and mitochondrial DNA, we postulate that the protein levels of γ-tubulin might synchronize gene expression of mitochondrial-related genes with on-going biological processes, such as mtDNA replication. To elucidate the effect of *γTubulin* reduction on mitochondrial-related gene signatures, we made use of mitochondrial-regulating gene signatures from 58 pre-defined gene sets containing mitochondrial-associated genes (Supplementary Data 1). Also, we created a custom gene set using all genes in the MitoCarta^23^. The detailed listing of all 59 gene sets is presented in Supplementary Data 1. The effect of *γTubulin* reduction on mitochondrial target gene signatures were examined by performing a gene set enrichment analysis (GSEA)^24, 25^ between RNA-seq samples from MCF10A and from *γTubulin*sh-MCF10A cells. Among the top 10 gene sets found to be affected, we noted biological process such as regulation of mitochondrial membrane permeability and reactome RNA, POL I, RNA POL III, as well as mitochondrial transcription (Supplementary Tables 1 and 2). The detailed results of the GSEA that shows an example of an enrichment plot produced for one of the used gene set is presented in Fig. 5a and Table 1. In concert, the data presented here suggest that alterations in the γ-tubulin meshwork affect the expression of mitochondrial-related genes.

**Table 1:** Upregulated mitochondria-related genes

No.	Gene	P value	FDR	No.	Gene	P value	FDR
1.	GTPBP3	6.8 10^-14^	7.9 10^-10^	16.	FAM210A	2.6 10^-5^	9.6 10^-3^
2.	HTATIP2	2.0 10^-10^	7.2 10^-7^	17.	CMC2	2.9 10^-5^	1.0 10^-2^
3.	RECQL4	1.7 10^-9^	4.9 10^-6^	18.	SLC25A25	3.0 10^-5^	1.1 10^-2^
4.	EYA2	5.3 10^-9^	1.2 10^-5^	19.	UCP2	5.6 10^-5^	1.7 10^-2^
5.	COX5B	2.2 10^-8^	4.0 10^-5^	20.	OXLD1	1.3 10^-4^	3.2 10^-2^
6.	SLC25A6	3.1 10^-7^	3.3 10^-4^	21.	SLC25A29	2.2 10^-4^	4.9 10^-2^
7.	NME3	1.1 10^-6^	8.7 10^-4^	22.	CKMT1A	2.2 10^-4^	4.8 10^-2^
8.	TP73	3.2 10^-6^	2.0 10^-3^	23.	MPC1	1.7 10^-4^	4.1 10^-2^
9.	UQCRFS1	4.2 10^-6^	2.4 10^-3^	24.	HIST1H3J	2.9 10^-5^	1.0 10^-2^
10.	PMAIP1	4.9 10^-6^	2.7 10^-3^	25.	PINK1	2.3 10^-5^	9.0 10^-3^
11.	FAM195A	8.3 10^-6^	4.1 10^-3^	26.	AK2	1.8 10^-5^	7.5 10^-3^
12.	H2AFX	1.6 10^-5^	6.7 10^-3^	27.	CKMT1B	1.1 10^-5^	5.2 10^-3^
13.	BRF1	1,6 10^-5^	6.7 10^-3^	28.	HIST3H2BB	3.7 10^-7^	3.6 10^-4^
14.	FASN	1.8 10^-5^	7.4 10^-3^	29.	HIST1H2BJ	7.5 10^-8^	9.9 10^-5^
15.	MRPL43	2.0 10^-5^	7.8 10^-3^				

Genes are displayed in order of most positively enriched (genes 1–21) to the most negatively enriched (genes 22–29) in the *γTubulinsh*-MCF10A cells. *P* values and FDR values were produced using edgeR^53^, using a generalized exact binomial test

To further elucidate the effect of γ-tubulin on mtDNA dynamics, we analyzed changes in the expression of the mitochondrial-related genes *HTATIP2* (HIV-1 TAT-Interacting Protein 2) and *SLC25A6* (Solute Carrier Family 25 Member 6), and of the mitochondrial-encoded genes *MTCO2* and *ATP6* (ATP synthase F0 Subunit 6) in U2OS and MCF10A cell populations that stably expressing *γTubulin* shRNA. In comparison to control cells, reduced expression of γ-tubulin in *γTubulin*sh-U2OS and *γTubulin*sh-MCF10A cells caused an increased protein expression of MTCO2, HTATIP2 and SLC25A6, whereas the expression of ATP6 was decreased (Fig. 5b). Furthermore, it can also be noted that HTATIP2, and SLC25A6 were amongst the most positively enriched genes in *γTubulin*sh-MCF10A cells (Table 1). These observations confirm that the protein levels of γ-tubulin affect the expression of mitochondrial-related genes.

### The γ-tubulin meshwork controls mitochondrial function

To establish the metabolic effect of the interaction of mitochondria with γ-tubulin, we analyzed mitochondrial respiration by determining the cellular oxygen consumption rate with the Seahorse Extracellular Flux analyser (Methods section) in cells with variable concentrations of γ-tubulin. We measured the oxygen consumption rate in U2OS cells, *γTubulin*sh-U2OS and in *γTubulin*sh-U2OS cells stably co-expressing human GFP-tagged sh-resistant γ-tubulin (*γTubulin*sh-U2OS-γ-tubulin_resist_; Fig. 5c, Supplementary Fig. 5a, b).

Moreover, considering that the GTPase domain of β-tubulin affects microtubule dynamics^3, 26–28^, we postulated that the N-terminal region of γ-tubulin might control the γ-string meshwork associated with mitochondria, as it encloses γ-tubulin’s GTPase domain^11^. To test this, we mutated Cyst^13^ to an Ala (GFP-A^13^γ-tubulin_resist_), as this mutation impairs GTP binding to the GTPase domain^11, 28^ and stably co-expresses the mutated recombinant protein in *γTubulin*sh-U2OS cells (*γTubulin*sh-U2OS-A^13^γ-tubulin_resist_; Supplementary Fig. 5b). In addition, we monitored the oxygen consumption rate in citral dimethyl acetyl treated U2OS cells (CDA; impairs the GTPase activity of γ-tubulin^11^; Fig. 5c, d, Supplementary Fig. 5c). In comparison to control U2OS cells, only 100 µM CDA treatment significantly decreased the basal oxygen consumption rate per cell. Oligomycin, an inhibitor of the ATP synthase; FCCP, the uncoupler of mitochondrial oxidative phosphorylation carbonyl cyanide 4-(trifluoromethoxy) phenylhydrazone; and rotenone (interferes with the electron transport chain in mitochondria) had effects on the oxygen consumption rate in all the cell lines tested, by decreasing (oligomycin and rotenone) or increasing (FCCP) the oxygen consumption rate. However, neither oligomycin nor FCCP had an effect on the low basal oxygen consumption rate levels of CDA-treated U2OS cells (Fig. 5d). These data were further confirmed by a WST-1 assay, which measured the succinate-tetrazolium reductase system in the respiratory chain of the mitochondria, and confirmed that only CDA-treated cells exhibited an impaired mitochondrial respiratory capacity (Fig. 5e).

**Fig. 6:**
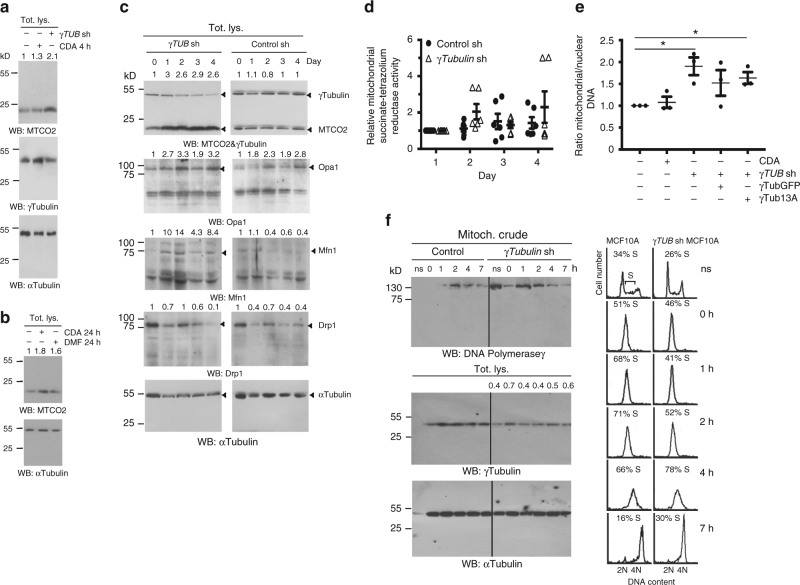
Reduced protein levels of γ-tubulin increase mitochondrial mass. **a**, **b** Total lysate (Tot. lys.) of U2OS cells, U2OS cells stably expressing *γTubulin* shRNA (*γTUB* sh) and U2OS treated with CDA or DMF for 4 h (**a**) or 24 h (**b**) before harvested. Protein levels of the mitochondrial marker MTCO2 and γ-tubulin were analyzed by Western blotting (WB) with an anti-MTCO2 and anti-γ-tubulin antibody, both originated in rabbit. Anti-α-tubulin was used as loading control (*N* = 4). **c** U2OS cells were transiently transfected with *γTUB* sh (day 0) or control shRNA (control sh) and changes in the protein levels of γ-tubulin, MTCO2, Drp1, Opa1 and Mfn1 and in the metabolic activity were analyzed by Western blotting (*N* = 3). **a**–**c** Anti-α-tubulin antibody was used as loading control. Numbers on the WBs indicate variations in MTCO2, Drp1, Opa1 and Mfn1 expression relative to day 0, as indicated. To adjust for differences in protein loading, the protein concentration of the various proteins was determined by their ratio with α-tubulin for each sample. The protein ratio in control extracts was set to 1. **d** WST-1 assay showing the metabolic activity of U2OS cells transfected with the indicated construct and treated as in **c** (mean ± SEM; *N* = 6). **e** Ratio between mitochondrial and nuclear DNA content measured by quantitative PCR in U2OS and U2OS cells stably expressing *γTub* sh and stably co-expressing GFP-γ-tubulin_resist_ (γTubGFP) or GFP-A^13^γ-tubulin_resist_ (γTub13A) and U2OS cells pre-treated with CDA for 4 h (mean ± SEM; *N* = 3, *P < 0.05; each sample the qPCR reaction was performed in triplicates). **f** Extracts from the mitochondria crude fraction from synchronous and non-synchronous (ns) MCF10A and MCF10A cells stably expressing *γTUB* sh were examined by WB using an antibody against DNA polymerase gamma (*N* = 3). DNA content was determined with a nuclear counter (percentage of S-phase cells indicated). The remaining mitochondrial crude depleted lysate (Tot. lys.) was run as loading control and examined with an anti-α-tubulin antibody. Numbers on the WBs indicate variations in γ-tubulin expression and are calculated as in **a**. Please see Supplementary Fig. 5

To investigate whether the contradictory effects of CDA treatment and *γTubulin*sh-U2OS and *γTubulin*sh-U2OS-A^13^γ-tubulin_resist_ cells depended on an adaptive mechanism of these cells to an impaired mitochondrial respiration, we estimated variations in the expression levels of the mitochondrial-encoded protein MTCO2 (Fig. 6a–c). CDA treatment did not affect the expression of MTCO2 most likely owing to the short-term exposure of the cells to the drug (4 h; Fig. 6a). However, longer exposure of U2OS cells to either CDA or to another inhibitor of the GTPase activity of γ-tubulin, dimethyl fumarate (DMF; Supplementary Fig. 5c)^11^ increased the expression of MTCO2 (Fig. 6b) and further confirmed that the γ-tubulin meshwork controls the expression of mitochondrial-related genes.

Accordingly, a transient *γTubulin* shRNA-mediated reduction of γ-tubulin protein levels caused an immediate increase of the levels of MTCO2 (Fig. 6c), whereas no significant changes of the mitochondrial capacity were observed (Fig. 6d). This agrees with a rapid adaptation of the mitochondrial respiratory capacity to variations in the γ-tubulin meshwork. Finally, the protein levels of optic atrophy 1 (OPA1, regulates mitochondrial shape and morphology) and mitofusin 1 (Mfn1, mediates mitochondrial fusion) were transiently increased after 1–2 days and were returned to normal levels after 4 days, whereas the protein levels of dynamin-related protein 1 (Drp1, mediates mitochondrial fission) were unaffected. This suggests that as adaptive changes are necessary for the adjustment of mitochondrial function and morphology to variations in the protein levels of γ-tubulin, the mitochondrial cellular mass adjusts (Fig. 6c).

### γ-Tubulin affects the replication of mitochondrial DNA

To confirm that changes in mitochondrial mass are an adaptive mechanism for the adjustment of mitochondrial function to variations in the protein levels of γ-tubulin, we estimated alterations in mtDNA copy number per cell by measuring the ratio between mitochondrial and nuclear DNA with quantitative PCR (Fig. 6e). Indeed, decreased levels of γ-tubulin resulted in increased amount of mtDNA in *γTubulin*sh-U2OS and *γTubulin*sh-U2OS-A^13^γ-tubulin_resist_ cells (Fig. 6e), confirming that to maintain a normal basal respiratory capacity, these cell lines need to adjust the mitochondrial mass (Fig. 5 and Fig. 6). However, inhibition of γ-tubulin with CDA did not affect the number of mitochondria, which is most likely due to the short time exposure of the cells to the drug (4 h; Fig. 6e).

Finally, to evaluate the effect of γ-tubulin protein levels on mtDNA replication, we altered γ-tubulin levels in a synchronized cell population. We found that in the crude mitochondria fraction of *γTubulin*sh-MCF10A cells, reduced protein levels of γ-tubulin had enhanced the protein levels of the mitochondrial-specific DNA polymerase gamma^29^ as the cells progressed through S-phase (Fig. 6f), which provides us with a potential molecular mechanism accounting for the observed rise in mtDNA.

### γ-*Tubulin* knockdown affects mitochondrial function

**Fig. 7:**
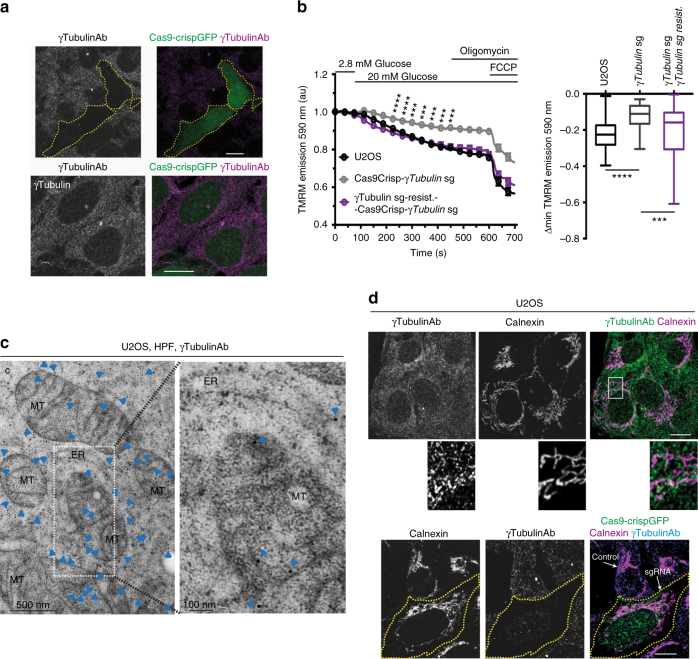
Sg-mediated knockdown of γ-*Tubulin* affects the activity of the mitochondria, but not the structure of the endoplasmic reticulum. **a** Confocal fluorescence microscopy of fixed U2OS stably expressing *γTubulin* sgRNA (Cas9-crispGFP) along or with a *γTubulin* sgRNA resistant gene (γTubulin). The protein levels of γ-tubulin in *γTubulin* sgRNA expressing cells were immunostained with an anti-γ-tubulin antibody (γTubulinAb) originating in mouse (*N* = 5). **b** U2OS were transfected with *γTubulin* sgRNA (Cas9Crisp-*γTubulin* sg) at day 0 and incubated for 7 days. Graphs show the mitochondrial membrane potential changes and the maximal hyperpolarization (maximal drop in emission) before oligomycin treatment (Δmin TMRM emission 590 nm) of single U2OS cells or single U2OS cells transiently expressing *γTubulin* sgRNA alone or with a *γTubulin* sgRNA resistant gene (γTubulin sg-resist.). The mitochondrial membrane potential was analyzed after addition of 20 mM glucose, 4 μg/ml oligomycin and 1 μM FCCP by recording the mitochondrial accumulation of the cell-permeant dye tetramethylrhodamine methyl ester (TMRM; mean ± SEM; unpaired two tailed Student’s *t*-test, Cas9Crisp-*γTubulin*sg vs. Cas9Crisp-*γTubulin*sg-γTubulin sg-resist, ***P < 0.001, ****P < 0.0001; U2OS, *N* = 107 cells; Cas9Crisp-*γTubulin*sg *N* = 46 cells; Cas9Crisp-*γTubulin*sg-γTubulin sg-resist. *N* = 128 cells). **c** Immunoelectron microscopy detection of endogenous γ-tubulin using an anti-γ-tubulin antibody that originated in rabbit, and gold conjugated protein A (γTubulinAb) in high-pressure frozen (HPF) U2OS cells. Images show the cytosol (C), mitochondria (MT) and endoplasmic reticulum (ER) of a U2OS cell. White dashed box shows the magnified area displayed in the inset (*N* = 5). **d** Confocal fluorescent microscopy of fixed U2OS or fixed U2OS transiently expressing *γTubulin* sgRNA (Cas9-crispGFP) that were immunostained with an anti-γ-tubulin (γTubulinAb) antibody that originated in rabbit, and with an antibody that recognized the ER marker calnexin (*N* = 5). Please, see Supplementary Fig. 4

In healthy mitochondria, the action of enzymes of the electron transport chain in the inner membrane of the mitochondria generates a proton gradient across the membrane, which drives the production of ATP. To determine whether endogenous γ-tubulin is essential for mitochondrial function, we measured in single cells the membrane potential by recording the accumulation of the cell-permeant dye tetramethylrhodamine methyl ester (TMRM). In cells with a polarized and intact inner mitochondrial membrane (∆ψ_m_), TMRM signal is bright, and the signal decreases upon hyperpolarization of the inner mitochondrial membrane, as a result of increased proton transport over the membrane. To examine these processes, we reduced the endogenous levels of γ-tubulin in U2OS cells by expression of *γTubulin* sgRNA (*γ*sgRNAU2OS). We added back γ-tubulin by stably co-expressing *γTubulin* sgRNA and a sg-resistant human *γTubulin* gene (*γ*sgRNAU2OS-γ-tubulin_resist_; Fig. 7a, Supplementary Fig. 4). In comparison to U2OS and *γ*sgRNAU2OS-γ-tubulin_resist_ cells, *γ*sgRNAU2OS cells responded to increased glucose levels by less polarization (lower decrease of signal; grey trace in Fig. 7b) of the inner mitochondrial membrane. This implies that the mitochondrial function in *γ*sgRNAU2OS cells is negatively affected in the absence of γ-tubulin. Inhibition of ATP synthase with oligomycin did not differentially affect the ∆ψ_m_ in any of the cells. These data confirm that γ-tubulin is necessary for robust function of the inner mitochondrial membrane. Similarly, the uncoupler of mitochondrial oxidative phosphorylation FCCP disrupted the ∆ψ_m_ of U2OS, *γ*sgRNAU2OS and *γ*sgRNAU2OS-γ-tubulin_resist_ cells equally (Fig. 7b), demonstrating that the mitochondrial transport and ATP phosphorylation are robustly coupled.

### The endoplasmic reticulum is not affected by γ-tubulin

Considering the regulatory role of γTURC’s association with the Golgi membrane-associated GMAP-210 protein in the proper positioning and biogenesis of the Golgi apparatus^30^, we investigated the possibility that γ-tubulin also interacts with and affects the morphology of the endoplasmic reticulum (ER). Immunoelectron microscopy of high-pressure frozen U2OS cells with an anti-γ-tubulin antibody showed that the anti-γ-tubulin antibody recognized the membrane of the ER (Fig. 7c). Immunofluorescent analysis of fixed U2OS cells co-stained with an antibody that recognized the ER marker calnexin and an anti-γ-tubulin antibody confirmed that the anti-γ-tubulin antibody recognized the membrane of the ER (Fig. 7d). To analyze the possible effects of the association between γ-strings and ER, we immunofluorescently co-stained endogenous γ-tubulin and the ER, in U2OS and *γ*sgRNAU2OS cells (Fig. 7d). We found that *γTubulin* sgRNA mediated depletion of γ-tubulin did not affect the morphology of the ER (Fig. 7d). Together these data suggest that γ-tubulin is associated with the endoplasmic reticulum, but these interactions do not have an impact on the morphology of the ER.

### The metabolite fumarate affects the γ-string meshwork

**Fig. 8:**
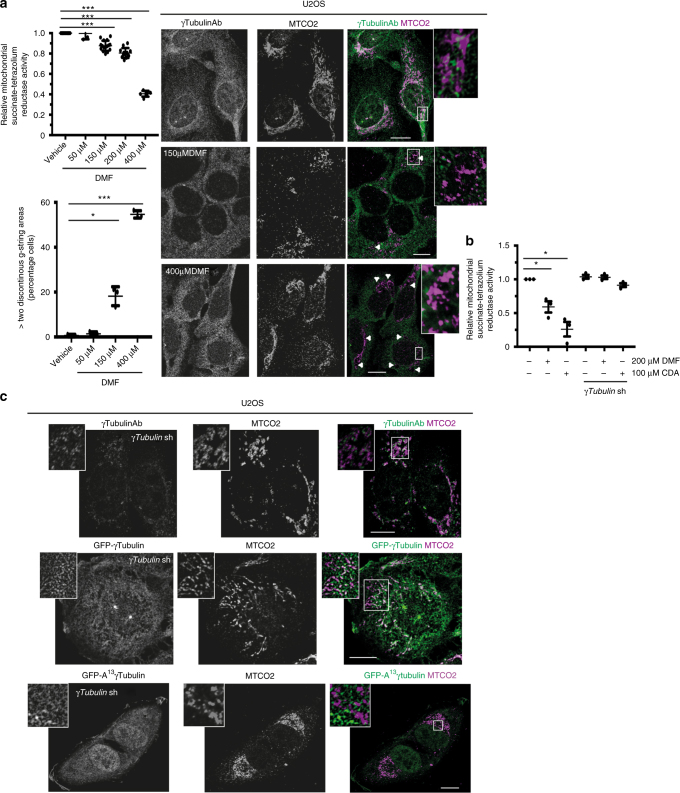
The cellular metabolite fumarate and γ-tubulin controls the shape of the mitochondrial network. **a** Confocal fluorescence images of fixed U2OS cells treated for 4 h with the indicated concentrations of DMF. The images show immunofluorescence stained endogenous γ-tubulin and MTCO2 using an anti-γ-tubulin (γTubulinAb) that originated in mouse, and an anti-MTCO2 antibody. Arrowheads and white boxes show cytosolic areas with discontinuous γ-strings and the magnified areas are displayed in the insets, respectively. WST-1 assay (relative mitochondrial succinate-tetrazolium reductase activity) showing the metabolic activity of DMF-treated U2OS cells (mean ± SD; *N* *=* 4–16, ***P < 0.001). The graph shows the percentage of cells with more than two areas containing discontinuous cytosolic γ-strings. A minimum of 100 cells was counted in each sample, and the percentage of cells was calculated (bottom; mean ± SD; *N* = 3, *P < 0.05, ***P < 0.001). **b** WST-1 assay showing the metabolic activity of DMF and CDA-treated U2OS and U2OS cells stably expressing *γTubulin* shRNA (mean ± SD; *N* = 3, *P < 0.05). **c** Confocal fluorescent microscopy of fixed U2OS cells that stably expressed *γTubulin* shRNA and co-expressed GFP-γ-tubulin_resist_ or GFP-A^13^γ-tubulin_resist_. The fluorescent images show representative areas of immunostained cells with an anti-γ-tubulin antibody, which recognized endogenous γ-tubulin and an endogenous anti-MTCO2, as indicated (*N* = 5). **a**, **c** The white boxes show the magnified areas displayed in the insets. Scale bars are 10 μm in images. Please, see Supplementary Figs. 5 and 6

A membrane-associated protein meshwork that regulates mitochondrial homeostasis may provide a cell with a tool to connect the cellular metabolic status with the mitochondrial respiratory chain. To test this hypothesis, we increased the endogenous levels of the metabolite fumarate by treating U2OS cells with DMF^11, 31^, a γ-tubulin inhibitor (Supplementary Fig. 5c) that is a cell-permeable derivative of fumarate. We then measured the activity of the succinate-tetrazolium reductase system in the respiratory chain of the mitochondria, as well as monitoring DMF’s effect on the mitochondrial network by immunofluorescence. Similar to CDA treatment, DMF impaired mitochondrial respiratory capacity in the treated cells (Fig. 8a). Moreover, the effect of both DMF and CDA on the mitochondrial succinate-tetrazolium reductase system was attenuated by reduced levels of γ-tubulin (Fig. 8b), which confirm that the effects of CDA and DMF on mitochondrial respiration are γ-tubulin dependent. Finally, immunofluorescent microscopy showed that the γ-tubulin inhibitor DMF disassembled γ-strings (Fig. 8a) and with the higher the amount of DMF, the more disassembled the γ-tubulin meshwork became (Fig. 8a). These findings provide a novel cellular mechanism that may synchronize the metabolic status of a cell with its mitochondrial activity.

We therefore investigated whether the γ-tubulin meshwork plays a role in the maintenance of the mitochondrial network. Similar to sgRNA and treatment with DMF (Figs. 2f and 8a), sh-RNAi-induced reduction of γ-tubulin disrupted the mitochondrial network, which became shorter and disorganized, in both *γTubulin*sh-U2OS and *γTubulin*sh-MCF10A cell lines (Fig. 8c, Supplementary Fig. 6a, b). This effect was reversed in U2OS cells by the expression of a shRNA-resistant GFP-γ-tubulin protein (Fig. 8c), but not by the expression of the A13γ-tubulin_resist_ GTPase mutant (Fig. 8c). Finally, treatment of U2OS cells with CDA also altered the mitochondrial network, which became disorganized (Supplementary Fig. 6c). These findings indicate that γ-tubulin expression and its GTPase domain are necessary for the organization of mitochondria in tubular structures.

### Transport to mitochondria affects the γ-tubulin meshwork

**Fig. 9:**
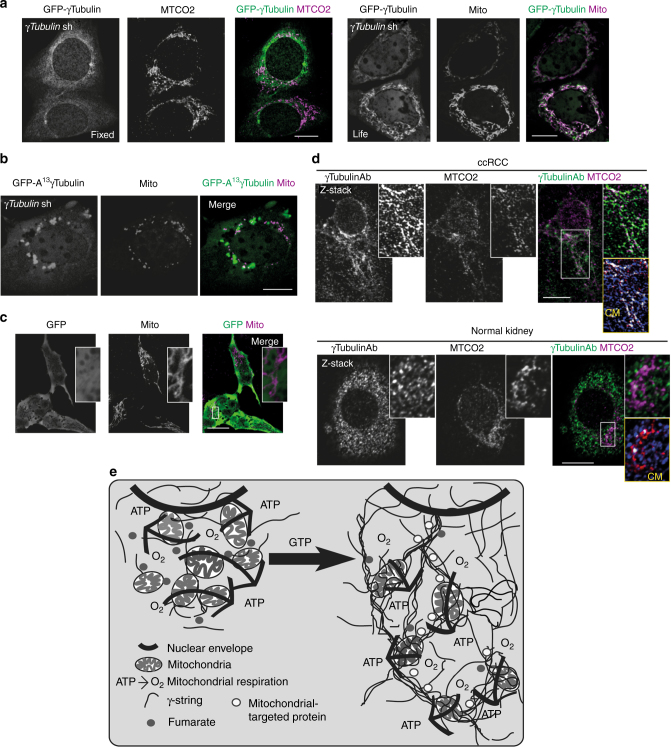
Increased mitochondria protein transport and low cellular mitochondria content affect the γ-tubulin meshwork. **a**–**c** Confocal fluorescent microscopy of fixed and live U2OS cells that stably expressed *γTubulin* shRNA and co-expressed the sh-resistant GFP-γ-tubulin_resist_ (**a**), the Cyst^13^ to Ala^13^ GFP-A^13^γ-tubulin_resist_ (**b**) or GFP (**c**) and transiently expressed pmTurquoise2-mito (mito), as indicated. Endogenous MTCO2 was stained with an anti-MTCO2 antibody (*N* = 3). **d** Average intensity projection of three Z-stack images (Z-stack) of fixed human clear cell renal carcinoma (ccRCC) and human normal kidney cells. Cells were imaged by confocal immunofluorescent staining with an anti-γ-tubulin antibody (γTubulinAb) that originated in mouse, and anti-MTCO2 antibody (*N* = 3). The yellow box shows co-localization pixel-map (CM) of the red and green (blue) channels of the magnified area displayed in the inset. White areas denote colocalized pixels between channels (ccRCC, Person’s *R* = 0.3, fraction of red (MTCO2) overlapping blue (γTubulinAb) M1 = 0.9, fraction of blue overlapping red M2 = 0.8; normal kidney, Person’s *R* = 0.12, M1 = 0.9, M2 = 0.7. **a**–**d** Scale bars 10 μm. **e** The GTPase domain of γ-tubulin is necessary for organizing a meshwork of mitochondria-associated strings that regulates mitochondrial respiratory capacity and gene expression and connects these organelles to the nuclear envelope. The metabolite fumarate and mitochondrial-targeted protein transport regulate the γ-tubulin meshwork. Please, see Supplementary Fig. 2

Consistent with a dynamic γ-tubulin meshwork, we saw that the γ-tubulin meshwork associated with mitochondria became more distinct upon increased transport to the mitochondria triggered by the transient expression of the mitochondrial-targeting signal enclosed in mito (Fig. 2e, Supplementary Fig. 2c), suggesting that an increased protein transport to mitochondria may trigger rearrangements in the γ-tubulin meshwork. To address this possibility, we ectopically expressed mito in *γTubulin*sh-U2OS-γ-tubulin_resist_ (Fig. 9a) and *γTubulin*sh-U2OS-A^13^γ-tubulin_resist_ (Fig. 9b) cells, or mito and GFP in U2OS cells (Fig. 9c). Live imaging of *γTubulin* sh-U2OS-γ-tubulin_resist_ and GFP-expressing U2OS cells transiently expressing mito showed that in all mito-expressing cells, only GFP-γ-tubulin_resist_ was associated with mito. (Fig. 9a, c). By contrast, we found that the mito localization became dispersed throughout the cells in 30% of *γTubulin*sh-U2OS-A^13^γ-tubulin_resist_ cells, which expressed a γ-tubulin mutant with a defective GTPase domain (Fig. 9b). These data show that increased protein transport to the mitochondria induces the enrichment of GFP-γ-tubulin at mitochondria in a GTP-dependent manner.

### The fewer mitochondria, the more pronounced the γ-tubulin tubules

To further characterize the function of the γ-tubulin meshwork, we hypothesized that in a cell population with a need to enhance its respiratory capacity due to low mitochondrial content, for example, the γ-tubulin meshwork could be utilized as a tool to optimize mitochondrial respiration. We subsequently examined primary human clear cell renal carcinoma (ccRCC), as those cells have low mitochondrial content, and normal human kidney cells^32^. Immunofluorescent staining revealed a clear accumulation of mitochondria in γ-tubulin enriched areas in ccRCC (Fig. 9d), indicating that low mitochondrial content affects the organization of the γ-tubulin meshwork. Together, these data demonstrate that γ-strings form a cellular infrastructure component that may provide the cell with a tool to synchronize cellular metabolism by modulating the mitochondrial respiratory capacity.

## Discussion

Although the functions of γ-tubulin have been extensively studied over the past decades, there are still functions that remain to be unravelled. Here, we show that γ-tubulin forms γ-strings and these are enriched between and in mitochondria. The γ-string meshwork interacts with both the mtDNA, and with the inner mitochondrial membrane protein MTCO2, and also affects the replication of mtDNA and expression of mitochondria-related genes. Manipulation of the cellular protein levels of γ-tubulin, or of γ-tubulin GTP binding by either CDA/DMF treatment, or by mutating Cys^13^ on its GTPase domain, disrupts the structure of the γ-tubulin meshwork^11^ and affects mitochondrial respiratory capacity and mass. As the γ-tubulin protein levels drop below 40 % in *γTubulin* sgRNA expressing cells, the mitochondrial membrane potential is impaired and finally the mitochondrial integrity is lost, subsequently realising cytochrome c. In contrast, increased transport to the mitochondria, the expression of the C-terminal DNA-binding domain of γ-tubulin or disruption of γ-tubulin’s NLS, relocates γ-tubulin to the mitochondrial meshwork. Moreover, the cellular mitochondrial content and the metabolite fumarate affect the γ-string meshwork. In view of these observations, we propose that the mitochondria-associated γ-tubulin meshwork provides mitochondria with a structural scaffold that regulates mitochondria function (Fig. 9e). Together, these data provide a novel mechanistic explanation on how the microtubule-independent functions of γ-tubulin affect the metabolic status of a cell.

Changes in mitochondrial network, mass and function occur in response to cellular stress. Fusion, fission and movement of mitochondria maintain the mitochondrial dynamics and morphology. In addition, formation of a mitochondrial network is important for maintaining mtDNA integrity and function^33^. In a previous study, the protein kinesin family member 5B (KIF5B) and polymerised microtubules were shown to mediate the formation of a dynamic mitochondrial network at the cellular periphery^34^. However, how the structure of the mitochondrial network affects the integrity of mtDNA is currently unknown. In this study, we demonstrate that γ-tubulin structurally organises the mitochondrial network and binds to mtDNA. Using the formation of mitochondrial γ-strings, γ-tubulin may link the structure of the mitochondrial network with mtDNA integrity, also potentially mediating the interactions of mitochondria with microtubules^17, 18, 34^.

*TUBG1* knockout mice survive only to morula/blastocyst stages, because a redundant function is activated by expression of *TUBG2* during the first stage of embryonic development^35^. In human neuroblastoma cell lines, both neuronal development and mitochondrial-induced oxidative stress results in upregulation of *TUBG2*, which is considered to be a pro-survival signal^13^. Here we show that *γTubulin* sgRNA mediated depletion of γ-tubulin protein kills cell lines, whereas cell lines stably expressing *γTubulin* shRNA survive with approximately 50% of the γ-tubulin pool, demonstrating an important role of γ-tubulin in cellular homeostasis and cell survival.

γ-Tubulin depletion in *Xenopus laevis* egg extracts prevents nuclear formation, as a border of γ-strings around chromatin is necessary for the formation of the nuclear membrane^5^. Furthermore, the C-terminal region of γ-tubulin contains the DNA-binding domain and stable expression of γ-tubulin^336-451^ co-localizes with the mitochondrial meshwork and nuclear compartment^9, 14^. Both the nuclear compartment and the mitochondria contain chromatin and a double membrane, so accordingly, γ-tubulin associates to both compartments. Taking into consideration that the maintenance and expression of mtDNA depends on the mitochondrial import of many nuclear-encoded proteins that control mitochondrial function^29^, we hypothesize that the accumulation of γ-strings at the nuclear and mitochondrial membranes may connect the cytosolic and DNA-associated γ-tubulin pools. This association may establish a path for transport of proteins to the mitochondria^29^ and for converting cytosolic signals into a gene response^5, 14, 36^.

Proliferating tumour cells have a reprogrammed cell metabolism to sustain cell growth and proliferation that is driven by genetic and non-genetic alterations. In the presence of oxygen, glucose is metabolized in the cytoplasm to pyruvate and then pyruvate is oxidized in the mitochondria^37^. Thus, cell metabolism requires the synchronous production and coordinated transport of metabolites between cell compartments that might be assisted by the γ-tubulin meshwork. Notably, in various tumours and cell lines, the localization and expression of γ-tubulin are altered^38–40^. Thus, the function of γ-tubulin as a regulator of the metabolic status of a cell may be one of various mechanisms that provide tumour cells with metabolic advantages that favour tumour growth.

Interactions between mitochondria and various cytoskeleton networks are known to influence mitochondrial respiration, morphology and cellular localization^18^. Nonetheless, to our knowledge there is no description of the cytoskeletal element that shapes the mitochondria network and synchronizes cytosolic and nuclear events with mitochondrial function. We therefore propose that a network of γ-strings forms a cytosolic meshwork that organises mitochondria and provides a structural basis for the mitochondrial machinery. In this model, the degree of association of γ-strings with mitochondria creates a cellular infrastructure component that may provide cells with a tool to synchronize the cellular metabolism with cellular function (Fig. 9e).

Our results demonstrate the existence and the regulation and function of a novel cytoskeletal element, the γ-string meshwork and provide a logical explanation for the mechanism underlying the location of mitochondria in close vicinity to the nuclear envelope. Furthermore, these findings uncover a novel regulatory mechanism that controls mitochondrial homeostasis.

## Methods

### cDNA and reagents

PmTurquoise2-tagged- mitochondrial-targeting signal from COX8A (amino acids 1–29; plasmid 36208; Addgene, Cambridge, MA, USA) was provided by Dr. D. Gadella^15^. Human pEGFP-γ-tubulin^334–449^ (γ-tubulin (334–449)), pEGFP- sh-resistant *TUBG1* gene, pEGFP-A13-γ-tubulin_resist_, and *γTubulin* shRNA were prepared as previously described^4, 11, 14^. pSpCas9(BB)-2A-GFP *γTubulin* sgRNA, and pcDNA3-sg-resistant *TUBG1* gene were prepared as previously reported^8^. The γ-tubulin fragment γ-tubulin (336-451) was prepared using a Quickchange Mutagensis Kit (Stratagene) and the primers listed in Supplementary Table 3. Finally, excision of the N-terminal region of γ-tubulin (γ-tubulin^336–451^) with *Hin*dIII was performed before re-ligation of the final construct. The Arg399-to-Ala–Lys400-to-Ala–Arg409-to-Ala substitutions in pcDNA3-sg-resistant *TUBG1* gene were prepared using a Quickchange Mutagensis Kit (Stratagene) and the primers listed in Supplementary Table 3. The mutations and constructs were verified by sequencing.

The following antibodies and reagents were used: anti-GFP (1:500), anti-GCP2 (1:500), anti-DRP1 (1:500), anti-Mfn1 (1:500), anti-OPA1 (1:500), anti-cytochrome c (1:400) and anti-calnexin (1:400, all from Santa Cruz Biotechnology, Dallas, USA); anti-γ-tubulin (1:500, mouse, T6557 recognizes the N-terminal amino acids 38 to 53 of γ-tubulin and rabbit, T3320, recognizes the C-terminal amino acids 437 to 451 of γ-tubulin, antibodies, Sigma-Aldrich, Saint Louis, USA), anti-α-tubulin (1:1000, Millipore, California, USA); anti-MTCO2, anti-HTATIP2, anti-ATP6, anti-SLC25A6, (1:400, Abcam, Cambridge, UK); anti-cox8 (1:500, Atlas antibodies, Stockholm, Sweden); anti-histone (1:400, Merck); MitoTracker Red CMXRos (Molecular Probes); citral dimethyl acetal and dimethyl fumarate (Sigma-Aldrich, Munich, Germany). All other reagents were obtained from Sigma-Aldrich.

Total lysates from cells, and Western blot analysis were prepared as reported^4, 14, 41^.

### Cell culture

Human osteosarcoma U2OS, human retinoblastoma Y79 and human mammary gland epithelia MCF10A cells were cultured as described^4, 11^. Stably or transient transfected *γTubulin* shRNA, pEGFP-γ-tubulin_resist_, pEGFP-A13-γ-tubulin_resist_, γ-tubulin_sgrest_, γ-tubulin^R399A-K400A-R409A^_sgrest_ and γ-tubulin^336–451^ U2OS, and MCF10A cells were obtained as described in Supplementary Table 4 and elsewhere^11^.

Primary human kidney epithelial and renal carcinoma cells were isolated after informed consent was obtained from participants. Primary cells were cultured from patient nephrectomies and subsequent analyses were performed in accordance with the ethical approval from Lund University ethical committee (LU680-08 and LU 289-07). An experienced pathologist classified tumours as ccRCCs. Normal samples were collected from healthy kidney cortex farthest from the tumour. Excised tissue was cut in pieces and incubated overnight with 300 U/ml Collagenase type I (Gibco) and with 200 U/ml DNAse I (Sigma) in full media. The following day, cells were collected and incubated for 5 min with trypsin. The remaining cell suspension was serially filtered through 40 µm and 20 µm filcons. Isolated cells were grown in DMEM high glucose supplemented with 10% FBS and 1% penicillin/streptavidin (Thermo Scientific). All cell lines were routinely tested for the presence of mycoplasma.

U2OS cells were transfected with sgRNA and examined on day seven or for the indicated period of time^9, 11^. Cas9-crispGFP expressing cells (GFP-expressing cells) were counted in a fluorescence microscope in each sample.

### Fluorescent imaging microscopy

U2OS cells were cultured on coverslips and fixed as described previously^9, 11, 42^. Coverslips were then incubated in PBS staining buffer (PBSB; PBS, 1% Fetal Calf Serum and 0.5% BSA) to prevent non-specific antibody binding. Cells were incubated (1 h) with primary antibody (in PBSB), washed with PBSB, and incubated with Alexa488-labelled, Cy3-labelled, Dylight-labelled or Alexa647-labelled secondary antibody (Jackson). Slides were washed and mounted in Vectashield with diamidino-2-phenylindole (DAPI; Vector laboratories, Burlingame, California) or in slowFade Gold reagent (super resolution, ThermoFisher Scientific). Cells were treated with DMF or CDA for 20 h before imaging. A minimum of 100 cells was counted in each sample, and the percentage of cells was calculated. Super-resolution images were captured with an ELYRA PS.1 SIM/PALM super-resolution structured illumination (SR–SIM; Carl Zeiss) with an alpha Plan-Apochromat ×100 NA 1.46 oil immersion objective. The SR–SIM performed a multiple image acquisition procedure with varying illumination patterns and then used Zen software to reconstruct the acquired images into one super-resolved image that had double the spatial resolution in compared to a wide-field image.

Confocal and fluorescence imaging were performed using a Zeiss LSM 700 Axio Observer microscope with a Plan-Apochromat ×63 NA 1.40 oil immersion objective. Sequential images were collected at 0.2-µm or 0.34-µm intervals. All images captured with the Zeiss LSM 700 Axio Observer microscope that are presented in this article were subjected to a rolling ball background subtraction (Fiji). Co-localization analysis, 3D projections, and processing of images were carried out with ImageJ (Fiji) software. The plug-ins “just another co-localization” in Fiji and “colocalization threshold” were used to determine co-localization between two channels^43^. In short, a cote’s background subtraction was applied^44^ before determining the Pearsons’s correlation coefficient (Pearson’s *R*), Manders split coefficients above cote’s threshold (Manders’ M1 & M2), and colocalized pixel maps.

Near simultaneous Cas9-crispGFP/DIC imaging sequences were collected and analyzed as described elsewhere^9, 11^. Time-lapse images were captured every 8 min.

### Cell fractionation, Immunoprecipitation and Western blot analysis

Purification of the crude mitochondrial fraction from MCF10A and U2OS cells (20 × 10^6^/sample) were prepared as follows. Cells were first lysed in buffer containing 0.1% triton X-100 (BADT^9^) and the resulting supernatant was the total cytosolic lysate. The remaining pellet containing the crude mitochondria fraction and nuclei was resuspended in 300 ml of cold BAD buffer and drawn slowly into a 21 g ½ needle and ejected with one stroke 10 times. The chromatin fraction was removed by centrifuged the nuclei at 1,700×*g* for 5 min at 4 °C. The resulting supernatant was pooled together with the total cytosolic lysate and further centrifuged at 10,000×*g* for 10 min at 4 ^o^C. The pelleted crude mitochondria fraction was resuspended in immunoprecipitation buffer (100 mM tris, pH 7.5, 300 mM NaCl, 2 mM dithiothreitol [DTT], 2 mM EGTA, 2 mM MgCl_2_, 1% Triton X-100, 250 mM phenylmethylsulfonyl fluoride [PMSF] and 100 mM Na_3_VO_4_, 0.5 mg/ml aprotinina, 0.5 mg/ml leupeptin and 0.5 mg/ml pepstatin) and further solubilized by a short sonication (3 × 5 s). The final extracts were thereafter divided into three samples and subjected to immunoprecipitation as described^9, 14^. Total cell lysates^41^, and the different immunoprecipitates were analyzed by Western blotting using MTCO2 as molecular marker for inner mitochondrial membrane, as described^41^.

For immunofluorescence staining, mitochondria were prepared as described above. The crude mitochondria fraction was resuspended in 300 µl network assembly buffer (NAB: 40 mM hepes, pH 7.2, 150 mM NaCl, 1 mM DTT, 1 mM EGTA, 1 mM MgCl_2_, 250 mM sucrose, 2 mM GTP, 250 mM phenylmethylsulfonyl fluoride (PMSF) and 100 mM Na_3_VO_4_, 0.5 mg/ml aprotinina, 0.5 mg/ml leupeptin and 0.5 mg/ml pepstatin) and incubated for 1 h at 22 °C. Network assembly was ended by addition of 3% formaldehyde. Immunostaining of assembled mitochondrial network were performed by first air-drying 10 µl of the network on a Superfrost Plus glass slide (Thermo Scientific, USA) and thereafter were permeabilized for 3 min with methanol/acetone (1:1; v/v) at -80 °C before immunofluorescence stained as described elsewhere^4^.

Purification of the mitochondrial fraction from MCF10A cells (20 × 10^6^/sample) was prepared as follows. To remove possible cytoskeletal elements attached to mitochondria, cells were pre-incubated for 15 min with culture medium containing 100 ng/ml colcemid and 5 µg/ml cytochalasin B (37 °C, 5% CO_2_)^4, 5, 8^. Thereafter, purification of the mitochondrial fraction by a Percoll density gradient centrifugation was prepared as described elsewhere^45, 46^, with the following modifications. Cells were lysed on ice in a dounce homogenizern in 1 ml cold homogenisation buffer (HB: 5 mM hepes, pH 7.2, 210 mM mannitol, 70 mM sucrose, phenylmethylsulfonyl fluoride (PMSF) and 100 mM Na_3_VO_4_, 0.5 mg/ml aprotinina, 0.5 mg/ml leupeptin and 0.5 mg/ml pepstatin). The cell debris, unbroken cells, and nuclei were removed by centrifugation (1300×*g* for 5 min at 4 °C). The nuclear membrane fraction was obtained by resuspending and preparing lysates of the first pellet as described elsewhere^14^. The obtained nuclear membrane fraction was lysed in sample buffer (SB)^41^. The total cytosolic homogenate was further centrifuged at 10,000×*g* for 10 min at 4 °C and the pellet containing the crude mitochondrial fraction was resuspended in HB supplemented with 1 mM EGTA (HBE) and layered on preformed gradient consisting of 2 ml 30% Percoll layered over 0.8 ml 50% Percoll in HBE buffer in a 4 ml centrifuge tube (Supplementary Fig. 3c). The gradient was subjected to ultracentrifugation for 15 min at 95,000×*g* in a swinging-bucket rotor. Mitochondrial and endoplasmic reticulum fractions were carefully collected with a needle and diluted with HBE buffer (1:5) followed by centrifugation at 17,000×*g* for 10 min. The final pellets were resuspended in SB. The microsome fraction was pelleted by centrifugation of the remaining total cytosolic homogenate at 100,000×*g* for 15 min. Both the final microsome pellets and the cytosolic supernatant were dissolved in SB. The purified fractions were boiled and analyzed by Western blotting.

Uncropped images of all Western blots presented in the study are shown in Supplementary Figs. 7–10.

### Electron microscopy

For high-pressure freezing, U2OS cells were seeded to 100% confluence onto carbon coated (10 nm) 6 mm sapphire discs (Leica) in 12-well dishes (Nunc). Cells were cryo-preserved with high-pressure freezing (HPM100, Leica) followed by freeze substitution (Leica AFS2, Leica) for 48 h at −90 degrees in Acetone with 0.1% Uranyl Acetate and embedded in Lowicryl with polymerization at -25 degrees for 48 h. The 60 nm sections were cut with a Leica Ultracut UC7 (Leica, Wienna, Austria) and collected on one whole formvar coated carbon grids and 200 mesh Nickel grids. The sections were pre-incubated for 30 min with pre-incubation buffer (50 mM glysine, 0.1% sodium borhydride NaBH_4_, 0.05 M Tris pH 7.4, 0.1% Triton), before incubation with polyclonal anti-γ-tubulin antibody (T3320) in TBST (0,05 M Tris pH 7.4, 0.1% Triton, 1% BSA) for 2 h at room temperature, followed by 1 h incubation with gold conjugated protein A (1:100, 10 nm gold; Agar Scientific, Essex, UK) in TBST. Final staining was performed with filtered 0.5% uranyl acetate for 10 min.

For preparation of the crude mitochondrial fraction, mitochondria were prepared as described above but in the absence of triton X-100. Pellets of mitochondria were embedded in low-melting point agarose and fixed 2% v/v glutaraldehyde in 0.05 M sodium phosphate buffer (pH 7.4). Samples were rinsed three times in 0.15 M sodium cacodylate buffer (pH 7.4) and subsequently post-fixed in 1% w/v osmium tetroxide and 0.05 M potassium ferricyanide in 0.12 M sodium cacodylate buffer (pH 7.4) for 2 h. The specimens were dehydrated in graded series of ethanol, transferred to propylene oxide and embedded in Epon according to manufacturer’s instructions. Sections were prepared as above and stained with uranyl acetate and lead citrate. High-pressure frozen and mitochondrial samples were examined with a Philips CM 100 TEM (Philips, Eindhoven, The Netherlands), operated at an accelerating voltage of 80 kV. Digital images were recorded with an OSIS Veleta digital slow scan 2k × 2k CCD camera and the ITEM software package.

### Chromatin immunoprecipitation and expression analyses

Chromatin immunoprecipitation (ChIP) was described elsewhere^14^. ChIPs were performed using rabbit polyclonal antibodies: a mixture (1:1) of anti-γ-tubulin T3320 and affinity purified anti-γ-tubulin T5192 (Sigma). Coprecipitated chromatin from U2OS cells was analyzed by PCR for the presence of mitochondrial DNA between the following base pairs: 1880–2186, 2423–2640, 10,654–10,854 and 15,102–15,370, with the oligos listed in Supplementary Table 3. Uncropped images of ChIP analysis presented in the Fig. 4b are shown in Supplementary Figures 8.

Alternatively, coprecipitated chromatin from S-phase synchronized MCF10A and MCF10A cells stably expressing γTubulin shRNA were sequenced using the Proton system (ThermoFisher) according to the manufactures. In brief, a DNA sample was fragmented using the S2 system from Covaris. End repair and adaptor ligation were performed by the AB Library Builder System (ThermoFisher). Samples were amplified according to the Ion Xpress™ Plus and Ion Plus Library Preparation for the AB Library Builder™* System* protocol and size selected with a target range of 220-310 bp (Blue Pippin™, Sage Science). Library size and concentration were assessed by a Bioanalyzer High Sensitivity Chip (Agilent Technologies) and by the Fragment Analyzer system (Advanced Analytical). Samples were pooled together in sets of two, followed by template preparation on the Ion Chef™ System using the Ion PI Hi-Q Chef Kit (ThermoFisher). Samples were then loaded on Ion PI™ v3 chips and sequenced on the Ion Proton™ System using the Ion PI™ Hi-Q Sequencing 200 Kit chemistry (200 bp read length, ThermoFisher).

Total RNA from MCF10A and *γTubulin*sh-MCF10A cells was prepared as previous described^4, 14^. Thereafter, 50 ng of total RNA was reverse transcribed according to Ion AmpliSeq™ Transcriptome Human Gene Expression Kit Preparation protocol (ThermoFisher). The cDNA was amplified using Ion AmpliSeq™ Transcriptome Human Gene Expression core panel (ThermoFisher) and the primer sequences were then partially digested. Adaptors (Ion P1 Adapter and Ion Xpress™ Barcode Adapter, Life Technologies) were then ligated to the amplicons. Adaptor ligated amplicons were purified using Agencourt® AMPure® XP reagent (Beckman Coulter) and eluted in amplification mix (Platinum® PCR SuperMix High Fidelity and Library Amplification Primer Mix, ThermoFisher) and amplified. Size-selection and purification was conducted using Agencourt® AMPure® XP reagent (Beckman Coulter). The amplicons were quantified using the 2100 Bioanalyzer® (Agilent) with High Sensitivity DNA Kit (Agilent.) Samples were then pooled, six per pool, followed by emulsion PCR on the Ion OneTouch™ 2 System using the Ion PI™ Hi-Q™ OT2 Kit (ThermoFisher). The pooled samples were loaded on Ion PI™ v3 chips and sequenced on the Ion Proton™ System using the Ion PI™ Hi-Q Sequencing 200 Kit chemistry (200 bp read length, ThermoFisher).

### RNA-seq and ChIP-seq analysis

RNA-seq reads were mapped to the reference genome (including the mitochondria genome) using TopHat^47^ (version 2.0.9, parameters: read mismatches: 2, -read gap length: 2, -max insertion length: 3, -max deletion length: 3). The python package HTSeq^48^ (version: 0.6.0) was used to produce the count table (a table counted number of reads mapped to each gene). We calculated the RPKM value for each gene based on count table using locally developed Perl code. Differential gene expression analysis was performed using edgeR^49^. An FDR cutoff of 0.05 was used to select significantly differentially expressed genes. ChIP-seq reads were mapped to the reference genome Bowtie^50^ (version 1.0.0, parameters: -m: 1). The mitochondria genome was downloaded from Ensembl (ftp://ftp.ensembl.org/pub/release-75/fasta/homo_sapiens/dna/) and included in the reference genome for mapping. Differential peak analysis was performed using the bggdiff tool as part of MACS2 ChIP-seq analysis package^20^ (version: 2.1.1, parameter: -g: 100, -cutoff: 3, -min len: 200, -depth1: 1, -depth2: 1).

Gene set enrichment analysis (GSEA) was performed using software obtained at http://software.broadinstitute.org/gsea/index.jsp, using 58 mitochondria-related gene sets provided in MSigDB (http://software.broadinstitute.org/gsea/msigdb/index.jsp) and a customized gene set constructed using MitoCarta^23^.

### Measurement of mitochondrial respiration and membrane potential (∆ψ_m_)

Measurement of oxygen consumption rate per cell was performed in a Seahorse XF24 Extracellular Flux Analyzer (SeaHorse Bioscience, North Billerica, MA, USA) according to the manufacturer’s instructions. In brief, before measurement the medium was changed to non-buffered XF Assay medium and cells were treated with 100 µM CDA or DMF for 2 h in a non-CO_2_ chamber after which the oxygen consumption rate was analyzed. Oxygen consumption rate was determined following injection of 4 mg/ml oligomycin (ATP synthase inhibition), 1 mM FCCP (uncoupler of mitochondrial oxidative phosphorylation; Abcam, Cambridge, UK) and 0.5 mM Rotenone (interferes with the electron transport chain in mitochondria by inhibiting the electron transferring activity of Complex I) according to instructions for the Cell Mito Stress Test (SeaHorse Bioscience). Each sample was run in four replicates.

For ∆ψ_m_ measurements, cells were pre-loaded with low K buffer (135 mM NaCl, 3.6 mM KCl, 1.5 mM CaCl_2_, 0.5 mM MgSO_4_, 0.5 mM Na_2_HPO_4_, 10 mM HEPES, 5 mM NaHCO_3_, pH 7.4) supplemented with 2.5 µM cyclosporin A (prevents permeability transition pore opening and dye leakage from mitochondria), 2.8 mM glucose and 100 nM tetramethylrhodamine methyl ester (TMRM; Invitrogen) for 2 h. After washing the cells once with low K buffer, cells were resuspended in low K buffer supplemented with 2.8 mM glucose. TMRM fluorescence measurements were performed using 543-nm excitation, 585-nm long pass emission filter settings on a Zeiss LSM510 inverted confocal fluorescence microscope. ∆ψ_m_ measurements were performed in quench mode^51^.

### Cellular thermal shift assay

To demonstrate that CDA and DMF directly bind to γ-tubulin we monitor the effect of the drugs on the thermodynamic stabilization of γ-tubulin upon ligand binding, as described elsewhere^52^. In brief, Y79 were treated with 100 µM CDA, 150 µM DMF or vehicle (DMSO) for 1 h. After removing the supplemented culture media, cells were resuspended in PBS supplemented with protease inhibitors (2 × 10^6^cells/ml) and heat-treated as described^52^. Cell lysates were prepared by freeze-thaw the cells three times. The resulting cell lysates were analyzed by Western blotting using an anti-γ-tubulin and an anti-α-tubulin antibody^41^.

### Proliferation assay and cell cycle analysis

Metabolic activity was analyzed with WST-1 cell proliferation assay (Roche) according to the manufacturer’s instructions. In short, 8 h before measurement, 100 μl of resuspended U2OS cells or U2OS cells transiently expressing human *γTubulin* shRNA or resuspended U2OS cells supplemented with either 100 µM CDA or various concentrations of DMF were plated at a concentration of 180 cells/μl in 96-well plates. 4 h before measurement, 10 μl of WST-1 reagent was added into each well, and the absorbance was measured at 450 nm using Fluostar omega Microplate Reader (BMG labtech).

For cell synchronization, cells were arrested at early S-phase by presynchronization with thymidine as previously described^9^ and released for different periods. Cells were ethanol-fixed and cell cycle progression was analyzed in a Nucleocounter^®^ NC-3000™ by measuring cell DNA content with diamidino-2-phenylindole (DAPI) as described by the manufacturer (ChemoMetec, Denmark). Cell cycle profiles were analyzed with FlowJo (Tree Star, Inc.).

### Statistical analysis

All data are expressed as ±SEM or SD, as indicated, and statistical significance of the differences between two groups or more was analyzed by paired Student’s *t*-test unless otherwise indicated (*P < 0.05, **P < 0.01, ***P < 0.001, ****P < 0.0001). Western blotting bands were quantitated with ImageJ software.

### Availability of data and material

Raw sequence data and processed files are publicly available at NCBI Gene Expression Omnibus (GEO) (http://www.ncbi.nlm.nih.gov/geo) under the following accession numbers: GSM2884568, GSM2884569, GSM2884570, GSM2884571, GSM2884576, GSM2884577, GSM2884578, GSM2884579, GSM2884584, GSM2884585, GSM2884586, GSM2884587, GSM2884588, GSM2884589, GSM2884590, and GSM2884591. All other data not present in the manuscript or supporting materials are available from the corresponding author upon request.

## Electronic supplementary material

Supplementary Information

Description of Additional Supplementary Files

Supplementary Data 1
