# Supplementary material for: The GTPase domain of gamma-tubulin is required for normal mitochondrial function and spatial organization
Source: Commun Biol. 2018 May 3;1:37. doi: 10.1038/s42003-018-0037-3 (PMC6123723; doi:10.1038/s42003-018-0037-3)
Supplement: Supplementary file 1 — Supplementary Information [file 42003_2018_37_MOESM1_ESM.pdf]

Gene set	Enrichment score	Normalized enrichment score	P value	FDR
GO REGULATION OF MITOCHONDRIAL MEMBRANE PERMEABILITY INVOLVED IN APOPTOTIC PROCESS	-0.66	-1.76	<2.2 10^{-16}	0.04
GO POSITIVE REGULATION OF MITOCHONDRIAL MEMBRANE PERMEABILITY	-0.64	-1.65	0.01	0.11
GO POSITIVE REGULATION OF MITOCHONDRIAL OUTER MEMBRANE PERMEABILIZATION INVOLVED IN APOPTOTIC SIGNALING PATHWAY	-0.55	-1.60	0.01	0.13
GO REGULATION OF MITOCHONDRIAL OUTERMEMBRANE PERMEABILIZATION INVOLVED IN APOPTOTIC SIGNALING PATHWAY	-0.53	-1.60	0.01	0.10
GO RELEASE OF CYTOCHROME C FROM MITOCHONDRIA	-0.57	-1.52	0.03	0.18
GO MITOCHONDRIAL MEMBRANE ORGANIZATION	-0.44	-1.48	0.01	0.22
GO REGULATION OF MITOCHONDRIAL DEPOLARIZATION	-0.58	-1.43	0.06	0.28
GO INTRINSIC COMPONENT OF MITOCHONDRIAL MEMBRANE	-0.45	-1.36	0.08	0.41
GO INTRINSIC COMPONENT OF MITOCHONDRIAL INNER MEMBRANE	-0.53	-1.36	0.12	0.37
GO APOPTOTIC MITOCHONDRIAL CHANGES	-0.43	-1.35	0.06	0.35

Supplementary Table 1. Gamma-tubulin expression significantly correlates with mitochondrial related process. The top 10 gene sets found to be enriched in samples prepared from *γTUBULIN* shRNA expressing MCF10A cells

Gene set	Enrichment score	Normalized enrichment score	P value	FDR
REACTOME RNA, POL I, RNA POL III, AND MITOCHONDRIAL TRANSCRIPTION	0.52	2.27	$<2.2\ 10^{-16}$	$<2.2\ 10^{-16}$
GO OUTER MITOCHONDRIAL MEMBRANE PROTEIN COMPLEX	0.57	1.44	0.10	0.40
GO NEGATIVE REGULATION OF RELEASE OF CYTOCHROME C FROM MITOCHONDRIA	0.44	1.28	0.14	0.61
GO MITOCHONDRIAL CALCIUM ION HOMEOSTASIS	0.49	1.27	0.18	0.47
GO MITOCHONDRIAL CALCIUM ION TRANSPORT	0.51	1.19	0.27	0.53
GO MITOCHONDRIAL ELECTRON TRANSPORT UBIQUINOL TO CYTOCHROME C	0.40	1.08	0.34	0.71
GO MITOCHONDRIAL PROTEIN COMPLEX	0.23	1.05	0.31	0.69
GO MITOCHONDRIAL ELECTRON TRANSPORT NADH TO UBIQUINONE	0.28	1.02	0.40	0.69
GO RESPONSE TO MITOCHONDRIAL DEPOLARISATION	0.21	0.91	0.73	0.88
GO MITOPHAGY IN RESPONSE TO MITOCHONDRIAL DEPOLARIZATION	0.21	0.91	0.74	0.79

Supplementary Table 2. Gamma-tubulin expression significantly correlates with mitochondrial related process. The top 10 gene sets found to be enriched in samples prepared from MCF10A cells

A. Primer used for mutagenesis, mutated or inserted bases underlined			
Gene	Mutation	Forward primer	Reverse primer
TUBG1	γ-tubulin$^{336\text{-}451}$	CACCCAGGTCCACAAGCTTGATGAGCTTGCAGAGGATCC	GGATCCTCTGCAAGCTCATCAAGCTTGTGGACCTGGGTG
TUBG1	R399A–K400A–R409A	5’CAGTATGACAAGCTGGCTGCGCGGGAGGCCTTCCTGG3’	CCAGGAAGGCCTCCCGCGCAGCCAGCTTGTCATACTG

B. Primer used for amplification		
Gene targeted	Forward primer	Reverse primer
γ-tubulin$^{336\text{-}451}$	GCGAAGCTTATGAGCTTGCAGAGGATCC	GCGGAATTCTCACTGCTCCTGGGTGCCCCAGGAGAT

C. Primer used for detection of mitochondrial DNA		
Amplified region	Forward primer	Reverse primer
1880-2186	GCAAGGAGAGCCAAAGCTAAG	CTTTTAGGCCTACTATGGGTG
2423-2640	CTGTCAACCCAACACAGGCATG	GAGCCATTCATACAGGTCCCT
10654-10854	TGCCATACTAGTCTTTGCCGC	GCTGTGGGTGGTTGTGTTGAT
15102-15370	CCTCCTGCTTGCAACTATAGC	GGTTGTTTGATCCCGTTTCGTG

Supplementary Table 3. Description of mutagenesis and primers used in this study

A. Modified cell lines				
Cell line	Expressing	Co-expressing	Selection	Denoted
U2OS	*γTUBULIN* sgRNA Cas9-crispGFP	–	GFP	*γTUBULIN* sgRNA
U2OS	*γTUBULIN* shRNA	–	Zeocin	*γTUBULIN*sh-U2OS
U2OS	*γTUBULIN* shRNA	GFP-γ-tubulin$^{334\text{-}449}$	Zeocin, G418	*γTUBULIN*sh-U2OS-γ-tubulin$^{334\text{-}449}$
U2OS	γ-tubulin$^{336\text{-}451}$	–	G418	γ-tubulin$^{336\text{-}451}$
U2OS	*γTUBULIN* sgRNA Cas9-crispGFP	γ-tubulin$_{sgresist.}$	GFP, G418	γ-tubulin$_{sgresist.}$
U2OS	*γTUBULIN* sgRNA Cas9-crispGFP	γ-tubulin$^{R399A\text{-}K400A\text{-}R409A}_{sgresist.}$	GFP, G418	γ-tubulin$^{R399A\text{-}K400A\text{-}R409A}_{sgresist.}$
MCF10A	*γTUBULIN* shRNA	–	Zeocin	*γTUBULIN*sh-MCF10A
U2OS	*γTUBULIN* shRNA	GFP-γ-tubulin$_{resist}$	Zeocin, G418	*γTUBULIN*sh-U2OS-γ-tubulin$_{resist}$
U2OS	*γTUBULIN* shRNA	GFP-A^{13}γ-tubulin$_{resist}$	Zeocin, G418	*γTUBULIN*sh-U2OS-A^{13}γ-tubulin$_{resist}$

Supplementary Table 4. Description of modified cell lines used in this study

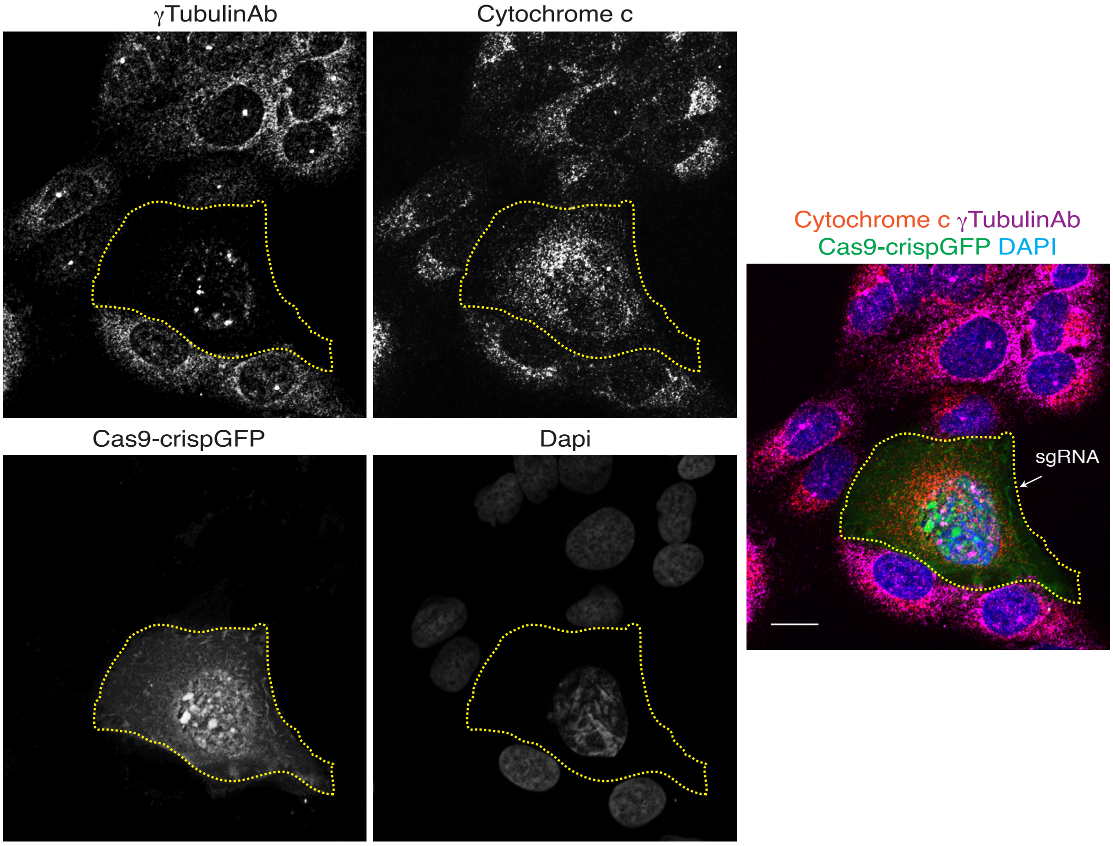

Supplementary Figure 1: γ-tubulin knockdown causes release of cytochrome c from the mitochondria. Images show average intensity projection of thirteen confocal Z-stack images of fixed U2OS cells transiently expressing *γTUBULIN* sgRNA (Casp9-crispGFP) that were immunostained with an anti-cytochrome c and an anti-γ-tubulin antibody originated in rabbit (γTubulinAb). The chromatin was stained with Dapi (N = 4). U2OS were transfected with *γTUBULIN* sgRNA at day 0 and incubated for 8 days before fixation.

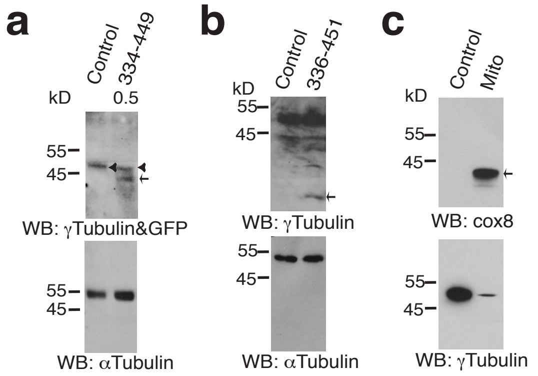

Supplementary Figure 2: Expression levels of various recombinant proteins in U2OS cells. (**a**) Western blots (WB) show total lysates of U2OS (Control) and U2OS cells that stably co-expressed GFP-γ-tubulin$^{334\text{-}449}$ (334-449) and *γTUBULIN* shRNA using a mixture (1:1) of an anti-γ-tubulin originated in rabbit and anti-GFP antibody. Numbers on WBs indicate the level of depletion of γ-tubulin in the extracts relative to control. To adjust for differences in protein loading, the protein concentration of γ-tubulin was determined by its ratio with α-tubulin for each sample. The protein ratio in control extracts was set to 1. (**b**) Total lysates of U2OS or γ-tubulin$^{336\text{-}451}$ (336-451) were analyses by WB with an anti-γ-tubulin originated in rabbit. (**a,b**) Anti-α-tubulin antibody was used as loading control (N = 3). Arrowheads and arrows indicate endogenous- and recombinant-γ-tubulin, respectively. (**c**) WBs show total lysates of U2OS and U2OS cells that transiently expressed mito using an anti-γ-tubulin originated in mouse and anti-cox8 antibody. Anti-γ-tubulin antibody was used as loading control (N = 3). The arrow indicates pmTurquoise2-mito.

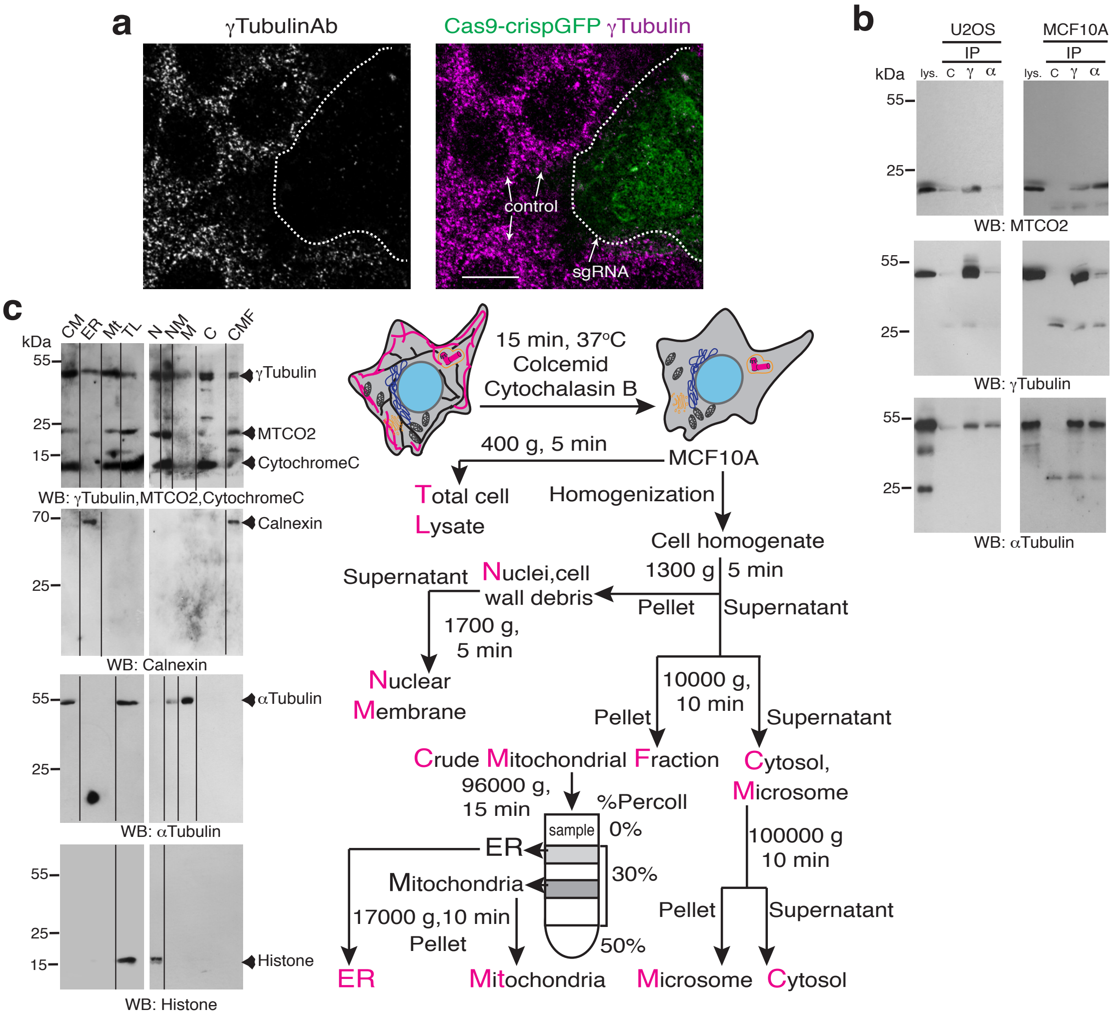

Supplementary Figure 3: Endogenous γ-tubulin associates with mitochondria. **(a)** Confocal fluorescence microscopy of fixed U2OS cells transiently expressing *γTUBULIN* sgRNA (Casp9-crispGFP) that were immunostained with an anti-γ-tubulin antibody originated in rabbit (γTubulinAb, red; *N* = 6). **(b)** U2OS and MCF10A cells (20 10^6 cells) were lysed and divided into three samples. Each sample was subjected to immunoprecipitations (IP) with an anti-γ-tubulin (γ) originated in mouse, an anti-α-tubulin (α) or an anti-GFP (C) antibody, as indicated, and developed by WB with antibodies against MTCO2, γ-tubulin (originated in rabbit) and α-tubulin. A total lysate was run as control (lys.) (*N* = 4). **(c)** Cells (20 × 10^6) were biochemically divided into the following cell fractions: cytosol/microsome (CM), endoplasmic reticulum (ER), mitochondria (Mt), total cell lysate (TL), nuclei and cell debris (N), nuclear membrane (NM), microsome (M), cytosol (C), and crude mitochondria fraction (CMF). Each fraction was examined by WB with first a mixture of an anti-γ-tubulin (originated in rabbit), an anti-MTCO2 (mitochondrial marker), and anti-cytochrome c (mitochondrial marker), followed by an anti-calnexin (ER), an anti-α-tubulin and anti-histone antibody (*N* = 4). Note that endogenous γ-tubulin is associated with the mitochondrial fraction. Flow scheme of the performed biochemical fractionation. The acronyms of the analysed fractions are labelled in magenta.

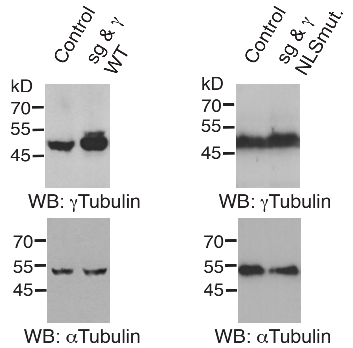

Supplementary Figure 4: Expression levels of various recombinant single-guided resistant proteins in U2OS cells. The expression of endogenous γ-tubulin (Control) and of the recombinant γ-tubulin$_{sgresist.}$ (sg & γ WT) and γ-tubulin$^{399\text{-}400\text{-}409}{}_{sgresist.}$ (sg & γ NLSmut.) proteins were analysed by WB in the total lysate from U2OS and U2OS cells stably expressing *γTUBULIN* sgRNA and co-expressing either γ-tubulin$_{sgresist.}$ or γ-tubulin$^{399\text{-}400\text{-}409}{}_{sgresist.}$ with an anti-γ-tubulin antibody originated in mouse. An α-tubulin loading control is shown (N = 3).

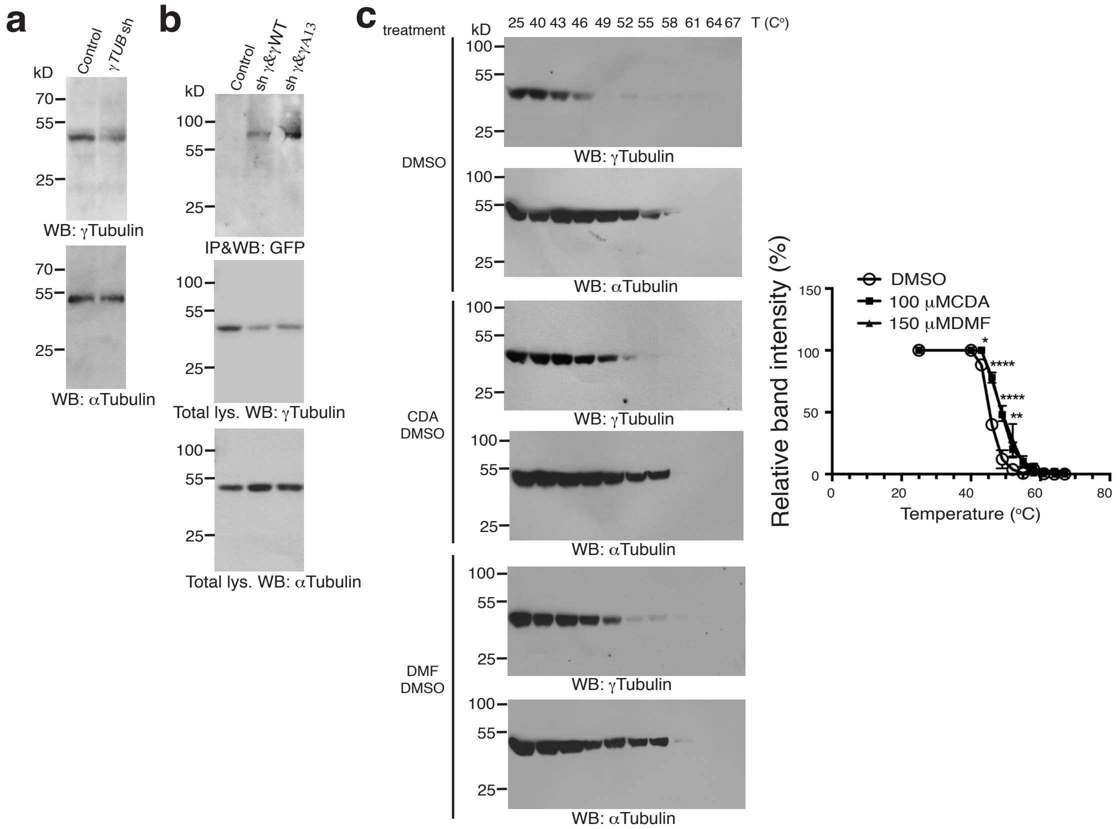

Supplementary Figure 5: CDA and DMF bind to γ-tubulin. (**a**) Total lysate of U2OS (Control) and of U2OS cells that stably expressed *γTUBULIN* shRNA (γ*TUB* sh) were analysed by western blotting (WB) with an anti-γ-tubulin antibody. An α-tubulin loading control is shown ($N = 6$). (**b**) U2OS or U2OS cells stably expressing *γTUBULIN*-shRNA (sh γ) and either GFP-γ-tubulin$_{resist}$ (γWT) or A^{13}-GFP-γ-tubulin$_{resist}$ (γA13), as indicated, were lysed. Each sample was subjected to immunoprecipitation (IP) and detected by western blotting (WB) with an anti-GFP antibody (GFP). Part of the lysate used for the immunoprecipitations was run as loading control (Total lys.) and the loading was analysed with an anti-α-tubulin antibody. (**c**) The cellular thermodynamic stabilization of γ-tubulin upon ligand binding were measured after 1 h pre-treatment of Y79 cells with DMSO, 100 μM CDA or 150 μM dimethyl fumarate (DMF). After heat-treatment of the pretreated Y79 cells, ligand-binding alterations in the heat-induced precipitation of γ-tubulin were monitored by western blotting analysis of total cell lysates with an anti-γ-tubulin antibody. To demonstrate the specific binding of CDA and DMF to γ-tubulin, an anti-α-tubulin antibody was used as control to show that the protein levels of α-tubulin were affected by neither CDA nor DMF pre-treatment, as indicated. Graph shows changes in the amount of cytosolic γ-tubulin after heat-induced precipitation as measured by densitometric analysis of the γ-tubulin protein content in the western blots membranes. Variations in the cytosolic protein levels of γ-tubulin were relative to the control (25 °C) that was set as 100 % ($N = 3$).

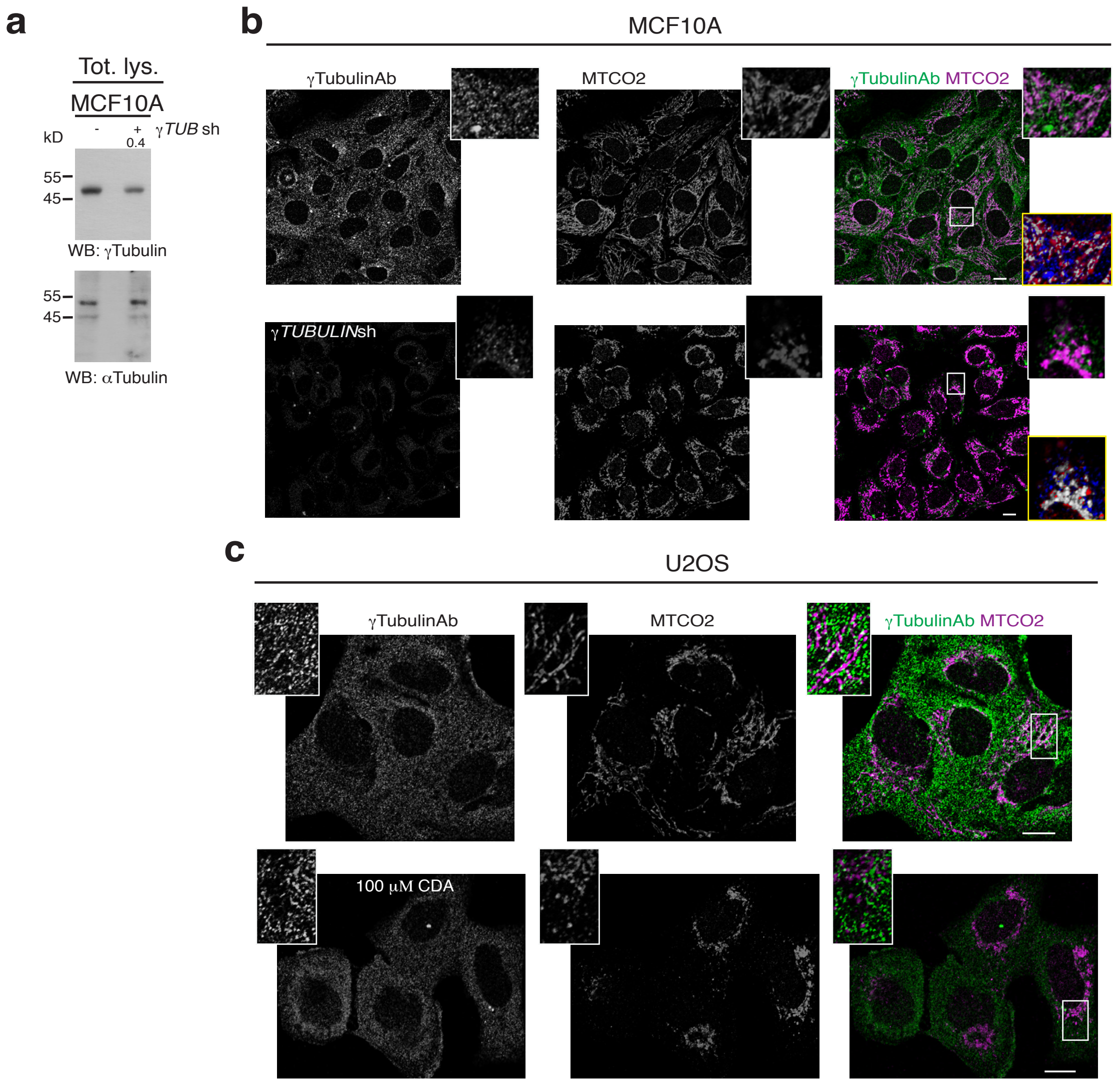

Supplementary Figure 6. Endogenous γ-tubulin structures the mitochondrial network. (**a**) Total lysate (Tot. lys.) of MCF10A cells that stably expressed *γTUBULIN* shRNA (γ*TUB* sh) were analysed by western blotting (WB) with an anti-γ-tubulin antibody. An α-tubulin loading control is shown ($N = 3$). Numbers on WBs indicate the level of depletion of γ-tubulin in the extracts relative to control. To adjust for differences in protein loading, the protein concentration of γ-tubulin was determined by its ratio with α-tubulin for each sample. The protein ratio in control extracts was set to 1. (**b**) Confocal fluorescence microscopy of fixated MCF10A and *γTUBULIN*-shRNA-MCF10A cells. The fluorescence images show representative images of immunostained cells with an anti-γ-tubulin antibody, which recognized endogenous γ-tubulin (γTubulinAb), and an anti-MTCO2 antibody, as indicated. The yellow boxes show colocalization pixel-maps (CM) of the red and green (blue) channels of the magnified area displayed in the inset. White areas denote colocalized pixels between channels. (**c**) Representative confocal fluorescence images of the effect of CDA on the mitochondrial network of fixed U2OS cells after 2 h treatment (bottom) compared to non-treated mitochondrial network (top). U2OS cells were immunostained with an anti-γ-tubulin originated in mouse and an anti-MTCO2, as indicated. **(b,c)** The white boxes show the magnified areas displayed in the insets ($N =$ 3). Scale bars are 10 μm in images.

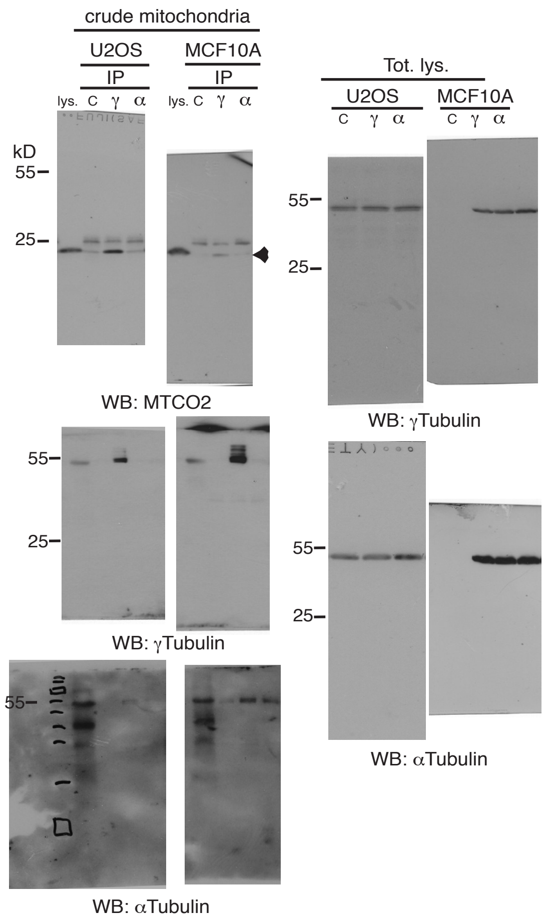

Supplementary Figure 7. Unedited western blots presented in **Figure 3c**. For more information see the main text.

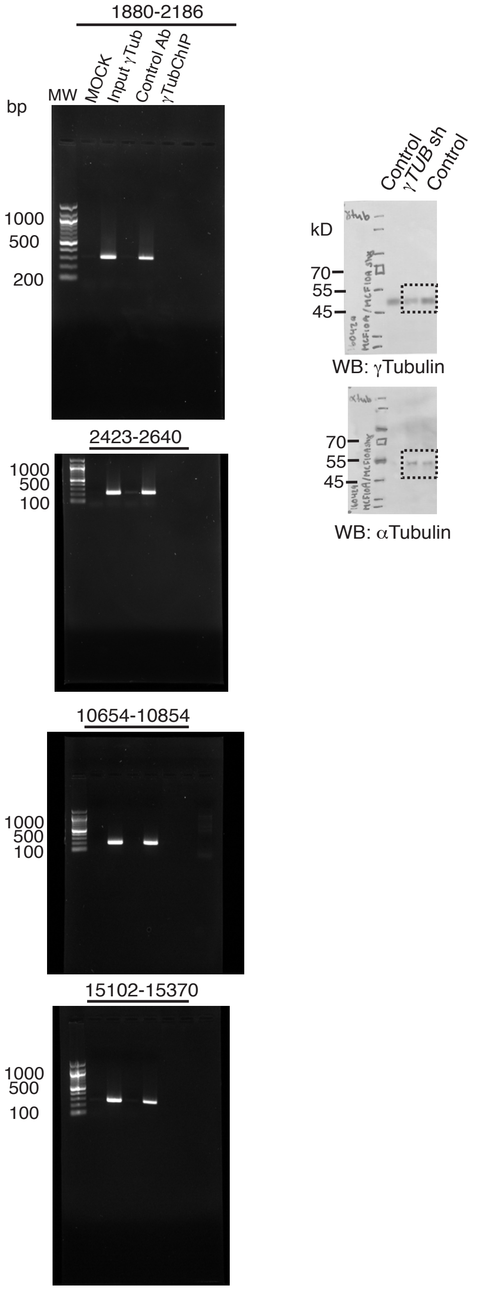

Supplementary Figure 8. Unedited gels and western blots shown in **Figure 4b** and **4c**, respectively. The black boxes display the samples shown in **Figure 4c**. For more information see the main text.

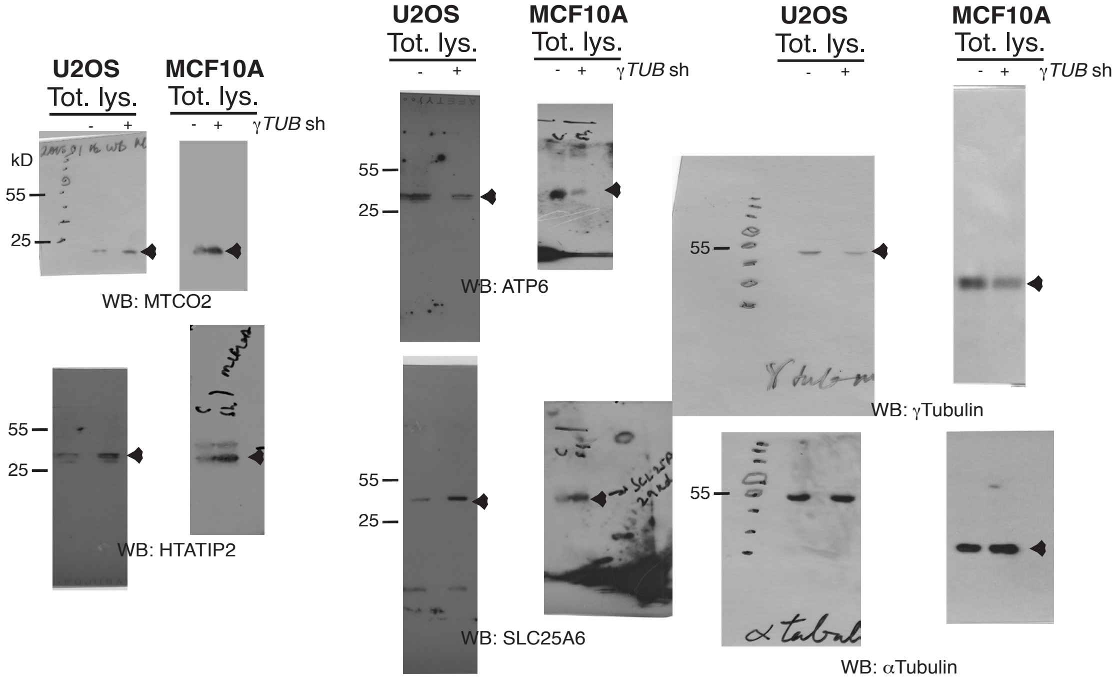

Supplementary Figure 9. Unedited western blots presented in **Figure 5b**. For more information see the main text.

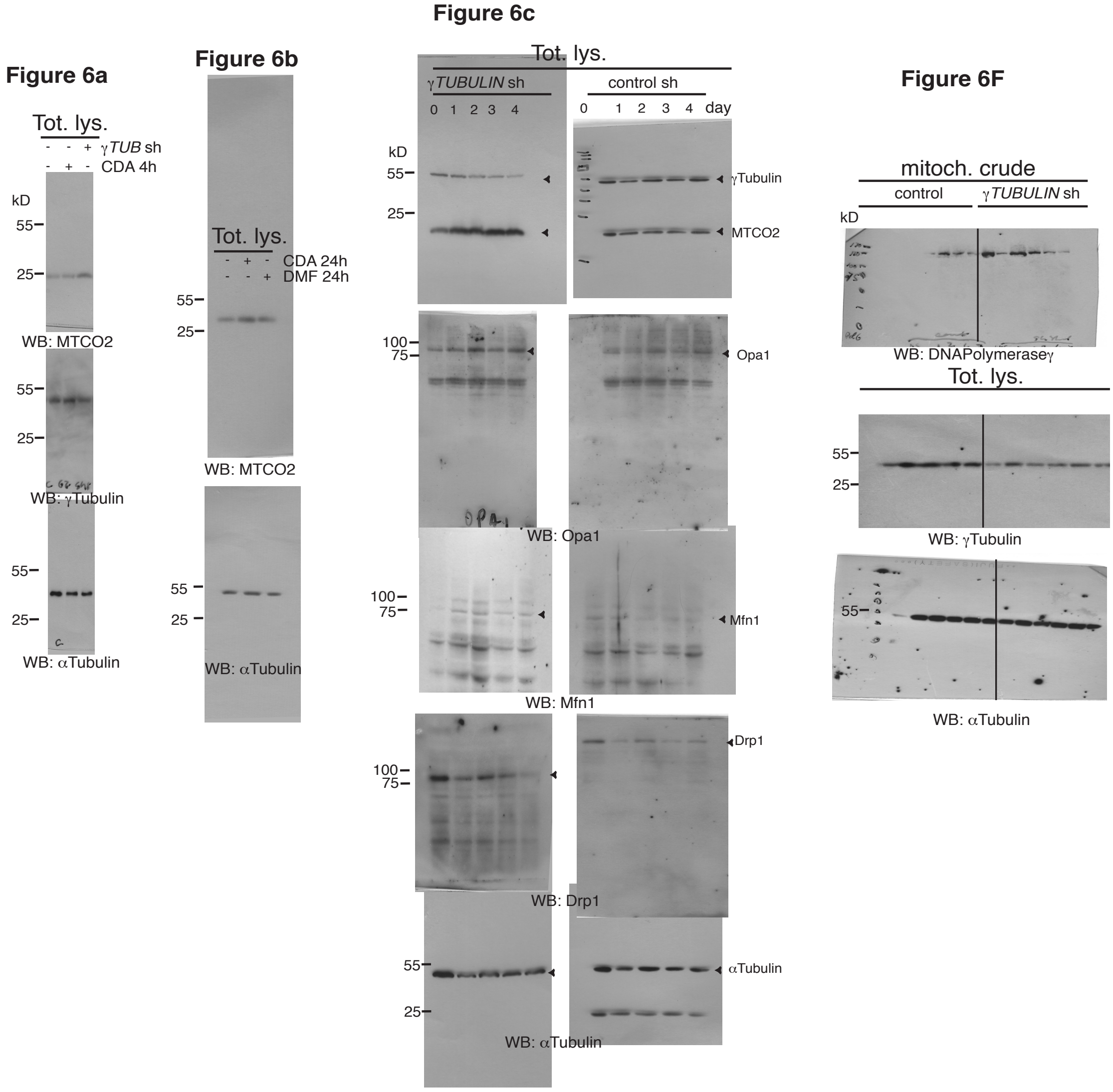

Supplementary Figure 10. Unedited western blots presented in **Figure 6a**, **6b**, **6c**, and **6f**, as indicated. For more information see the main text.
