# Supplementary material for: The GTPase domain of gamma-tubulin is required for normal mitochondrial function and spatial organization
Source: Commun Biol. 2018 May 3;1:37. doi: 10.1038/s42003-018-0037-3 (PMC6123723; doi:10.1038/s42003-018-0037-3)
Supplement: Supplementary file 2 — Description of Additional Supplementary Files [file 42003_2018_37_MOESM2_ESM.docx]

**Description of Additional Supplementary Files**

File Name: Supplementary Data 1

Description: Detailed listing of all genes included in the 59 gene-sets containing mitochondrial-regulating gene signatures.
